# Pole-to-pole LOV-domain receptor diversity points to an early stramenopile origin and global dominance of aureochromes

**DOI:** 10.1038/s42003-026-11067-1

**Published:** 2026-10-31

**Authors:** Melissa Misir, Peter G. Kroth, Sylke Wohlrab

**Affiliations:** 1 Plant Ecophysiology, Department of Biology, University of Konstanz, Konstanz, Germany; 2 Alfred Wegener Institute, Helmholtz Centre for Polar and Marine Research, Bremerhaven, Germany

**Keywords:** Molecular ecology, Molecular evolution

## Abstract

Light regimes are a fundamental environmental cue for marine organisms. Yet molecular adaptations of photoreceptors to the extreme seasonality of polar regions remain poorly understood. We demonstrate that the diversity of candidate blue-light-sensing LOV-domain proteins increases at higher latitudes. We identify aureochromes, blue-light-sensitive transcription factors unique to stramenopiles, as the most frequent LOV-domain receptors across ocean latitudes. Phylogenetic reconstructions reveal early aureochrome diversification in the stramenopile lineage, originating in a heterotrophic ancestor before photosynthesis was acquired. This challenges the assumption that aureochromes are restricted to phototrophs, suggesting an ancestral role in spectral light signaling. We find that, despite changes in light quality and photoperiod by latitude, diatom aureochrome domain architecture remains conserved. This implies adaptation through regulatory tuning, such as altered expression dynamics or post-translational control. Our results emphasise the importance of spectral-light sensing in polar oceans and link the widespread dominance of aureochromes to their ancient origin and conservation.

## Introduction

The fact that blue wavelengths penetrate deeper into seawater than any other wavelength in the visible spectrum characterizes the appearance of our blue planet. For marine organisms, blue light provides reliable information about depth, turbidity, and diurnal and annual rhythmicity, and has therefore become a central cue that regulates essential physiological and behavioral processes^1^. Moreover, the underwater light field fuels photosynthetic organisms, which constantly have to adjust to changes in light quality and quantity due to, e.g., mixing or light-absorbing particles^2,3^. In addition to the ability to quantify light by absorbing it through the photosynthetic machinery, phytoplankton species have specific photoreceptors that recognize a defined range of wavelengths and light intensities in the water column^3^.

The dominance of blue light in the water column may explain why algae, including phytoplankton, possess more algal-specific blue light photoreceptors than land plants^4^. Likewise, the phylogenetically diverse group of photosynthetic stramenopiles harbors a distinctive class of blue-light receptors referred to as aureochromes (AUREOs)^5^. AUREOs contain a DNA-binding bZIP-domain in the N-terminal region of the protein, followed by the C-terminal light-sensing flavin-binding LOV-domain (Light-Oxygen-Voltage domain). This architecture makes AUREOs blue light-sensitive transcription factors. In contrast to other photoreceptors that, after activation, initiate a signaling cascade, AUREOs, after activation, directly interact with promoters, allowing for very rapid responses^5–7^. Gene regulation occurs after dimerization of the bZIP-domains of two AUREO proteins and binding to promoters of target genes by a blue light stimulus^8^.

AUREOs have been particularly well-studied in diatoms, which are photoautotrophic algae belonging to the stramenopiles, and are part of the SAR clade (stramenopiles, alveolates, rhizarians). Within the stramenopiles, two major groups exist, the Gyrista and Bigyra. The Bigyra group only harbors species that are heterotrophic, while Gyrista consists of Pseudofungi (Oomycetes and Hyphochytriomycetes) and the photosynthetically active Ochrophytes^9^. So far, four paralogues of AUREO proteins have been described in the model diatom *Phaeodactylum tricornutum*: AUREO 1a, 1b, 1c, and 2^10^. The expression of some AUREOs (PtAureo1a and 1c) shows a diurnal pattern, whereas the expression of PtAureo1b is light-induced, while PtAureo2 expression is only weakly regulated^8^. Transcriptomic studies of *P. tricornutum* revealed that PtAureo1a is involved in regulating the cell cycle, light acclimation, the diatom clock^11^, and overall regulation of gene transcription^7,12^. Besides AUREOs, stramenopiles, like other eukaryotic algae, possess additional well-described photoreceptors such as the red- and far-red-light-absorbing phytochromes^13,14^, blue light-absorbing cryptochromes, blue light photoreceptors that are involved in photoprotection and circadian regulation, as well as a couple of further photoreceptors^15,16^.

Several proteins of unknown function have been identified in marine phototrophic organisms that possess a LOV-domain in combination with another domain^17^. One example are phototropins, blue light photoreceptors found in green algae and land plants, which combine two LOV-domains with a kinase domain. Further proteins show combinations like LOV/Ef-hand, LOV/HSF (heat shock factor), and RGS/LOV-domains (Regulator of G protein Signaling). Although LOV-domains can perceive different cues, the high combinatory number of LOV-domains fused to other domains indicates that marine unicellular planktonic organisms may harbor a diversity of possible light-sensitive regulatory mechanisms to thrive in changing light environments^17^.

While AUREOs from a few model organisms, like *P. tricornutum* or *Vaucheria frigida*, that live in non-polar regions have been studied in detail^5,7,18^, much less is known about AUREOs from polar regions^3^. In polar regions, apart from low temperatures, light quality and quantity conditions can be very extreme. Light conditions vary from day/night cycles to seasonal cycles, with polar nights as an extreme form of light shortage and continuous summer daylight as the opposite^19^. The fact that, for example, diatoms are nevertheless prevalent in these regions^20^, indicates that they have successfully adapted to respective conditions.

In this study, we show that global light regimes are associated with pronounced patterns in the diversity and evolution of LOV-domain containing proteins in marine unicellular eukaryotes. Our pole-to-pole metatranscriptomic analysis shows that LOV-domain richness increases towards higher latitudes, with AUREOs emerging as the most frequently detected LOV-domain receptor family across the global ocean. Phylogenetic reconstruction reveals an early origin and diversification of AUREOs within stramenopiles, including their presence in early-diverging heterotrophic lineages. Despite contrasting light regimes across latitudes, we detect no major amino acid differences between polar and non-polar AUREOs in either the LOV or bZIP domain. Together, our findings reveal strong latitudinal patterns in LOV-domain diversity while highlighting the widespread and conserved nature of AUREOs across global light regimes.

## Results

### Latitudinal patterns of LOV-domain richness and AUREO diversification across stramenopiles

**Fig. 1:**
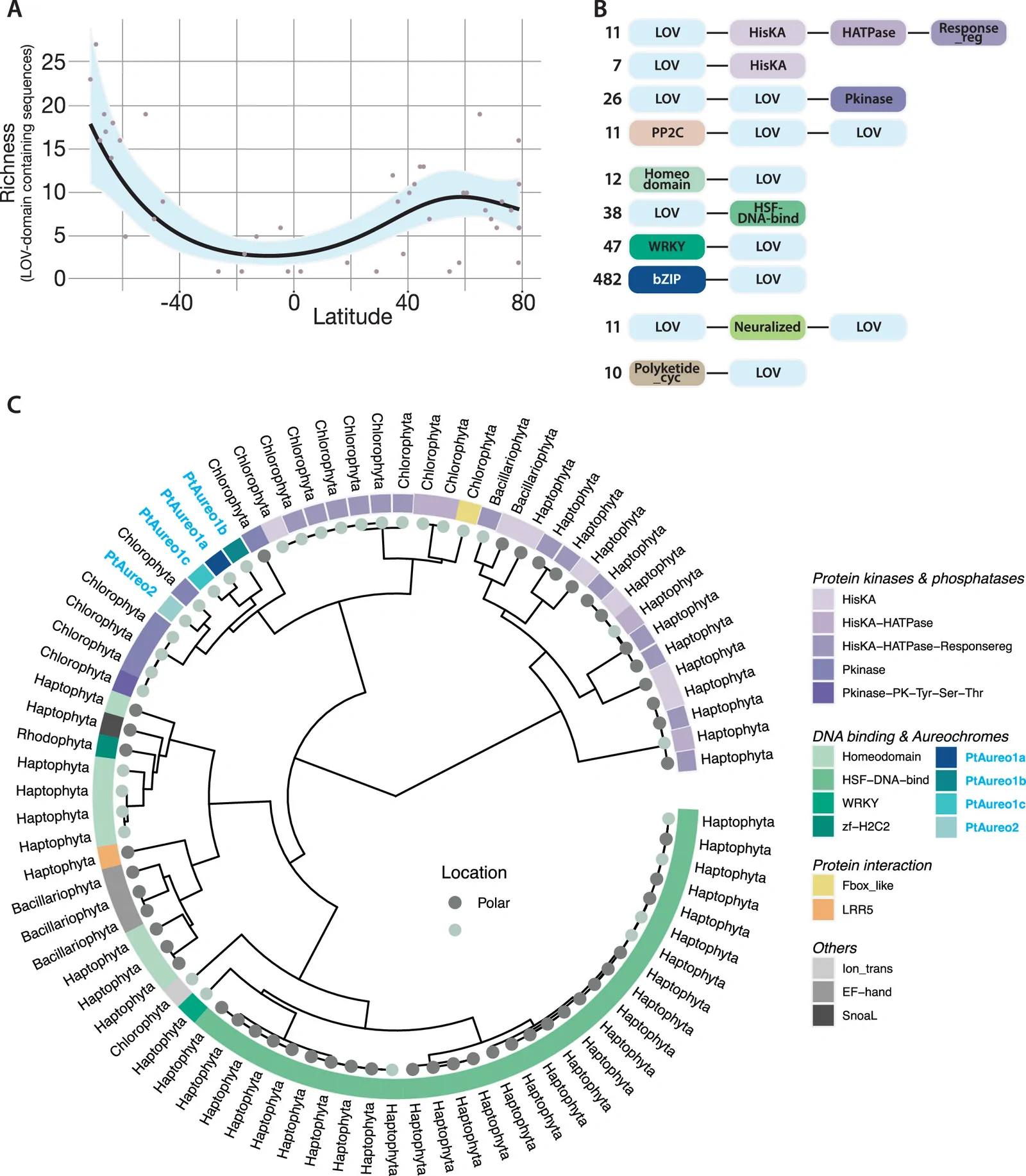
Analysis of metatranscriptomes focusing on transcripts encoding LOV-domains. **A** Richness of LOV-domain-containing proteins across the latitudes of the globe. The points represent calculated richness values per metatranscriptome; the black GAM line, including 95% confidence intervals, represents model-predicted LOV-domain richness across latitude while accounting for order-level Shannon diversity and sequencing depth. **B** Overview of the top 10 most frequently found LOV-domain combinations of the whole metatranscriptomic dataset; numbers are detection frequencies. **C** Profileview-tree of the LOV-domain of LOV-domain-containing genes in combination with other domains that could be taxonomically assigned within the eukaryotes from the pole-to-pole metatranscriptome dataset with *Phaeodactylum tricornutum* AUREOS as reference (PtAUREOs). Inner circle: Polar or non-polar sequence; outer circle: Domains found in combination with a LOV-domain.

Metatranscriptomic data from the “Sea of Change” project were analyzed with respect to the protein diversity of LOV-domain-containing proteins, as well as the respective domain architecture (Supplementary Data 1). Sequences from the Arctic, Antarctic, and non-polar regions were analyzed and then divided according to their origin above or between the polar circles (66.565° for the Arctic and 66.33° for the Antarctic). The area between the northern and the southern polar circles represents the non-polar region (Supplementary Fig. 1). LOV-domain richness was higher in samples from the polar oceans and showed a significant nonlinear relationship with latitude (negative-binomial GAM, *p* < 0.05, Fig. 1A). The latitudinal pattern represents richness-corrected values after accounting for order-level Shannon diversity and sequencing depth. The observed pattern indicates a possibly more important role of blue-light sensing elements in polar regions that are characterized by lower solar elevation angles and polar light conditions. We generally detected a large variety of LOV-domain proteins with different additional functional domains. The combination of a C-terminal bZIP-domain with an N-terminal LOV-domain, indicative of AUREOs, was found to be the most frequent LOV-domain combination, followed by LOV-domains either linked to a Histidine Kinase domain, an HATPase_c domain, a WRKY domain, or an HSF-DNA-binding domain (Fig. 1B).

To further classify the LOV-domains of the detected proteins, we constructed a tree representing clusters of LOV-domain sequences based on their similarity in sequence space using Profileview^21^ (Fig. 1C). Only eukaryotic sequences that could be further classified at the phylum level (based on the MMseq2 classification) were included in the dataset (Fig. 1C). In the case of proteins with more than one LOV-domain, e.g., phototropins, the respective LOV-domains were analyzed separately before constructing the sequence space tree. The obtained tree distinguished between LOV-domain proteins combined with diverse protein kinases and those having DNA-binding domains like AUREOs (Fig. 1C). However, the LOV-domains of the AUREOs clustered with the LOV-protein kinases rather than with other DNA-binding domain proteins.

**Fig. 2:**
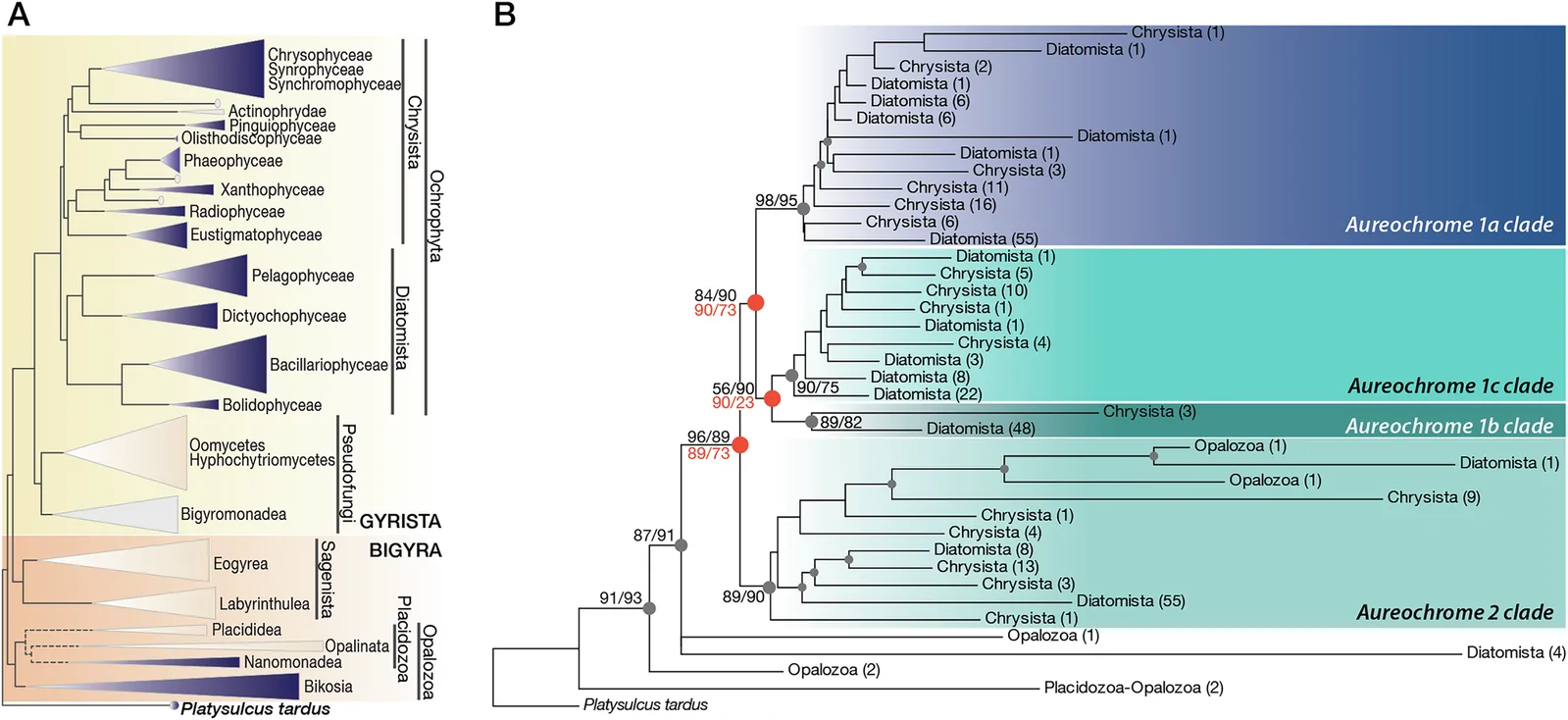
AUREO and early-diverging AUREO sequences within the stramenopiles. **A** Phylogenetic tree adopted from Cho et al.^69^, showing the major taxonomic clades. The dark blue clades contain AUREOs (isoforms 2, 1a, 1b, 1c) and early-diverging AUREO sequences; the light brown clades contain no AUREOs, and no reference data were checked for the gray clades. **B** Maximum-likelihood phylogeny of stramenopile AUREO sequences, including duplication retention patterns. Support values (gray, SH-aLTR/UFBoot, see “Methods”) are shown for all ancestral internal nodes that define the major, well-described AUREO clades. Red nodes highlight ancestral internal duplication events with high bootstrap reconciliation and retention support (as indicated by the red numbers). Gray dots within the AUREO clades mark nodes with SH-aLTR>70 and UFBoot>85; numbers in brackets correspond to the numbers of sequences within the respective clade. The unrooted version of the overall AUREO phylogenetic tree with all support values is provided in a supplementary file (Supplementary Data 6).

We screened 201 stramenopile protein datasets (*P. tardus*, 30 Bigyra, and 170 Gyrista), and retained 96 species (*P. tardus*, 5 Bigyra, and 91 Gyrista) with a total of 323 AUREO sequences (*P. tardus*, 7 Bigyra, and 315 Gyrista). Unexpectedly, two Bigyra lineages (Nanomonadea and Bikosia), which are entirely heterotrophic and lack plastids, apparently possess genes encoding AUREOs. Also, the recently discovered heterotrophic, deep-branching last common ancestor of the stramenopiles, *P. tardus*, possesses one gene encoding a potential AUREO protein (Fig. 2). Within the Bigyra, AUREOs seem to be absent in the Placididea, Opalinata (subgroups of the Opalozoa, Bigyra) and all Sagenista (Bigyra) lineages (Fig. 2A). Within the Gyrista, we could detect AUREOs in all major photosynthetic lineages (i.e. the Ochrophyta), while no genes were found that could be assigned to the non-photosynthetic Oomycetes and Hyphochytriomycetes (Fig. 2A). Our maximum-likelihood phylogeny of all identified AUREO sequences places almost all of them (313 out of 323) within the clades corresponding to the well-described AUREOs 2, 1a, 1b, and 1c isoforms. The 10 early branching AUREOs belong to *P. tardus*, the Bigyra lineage genera MAST3C, MAST3A, *Halocafeteria*, and *Cafeteria*, and the Gyrista genera *Octactis* (Dictyochophyceae), *Chrysosystis*, and *Aureoumbra* (both Pelagophyceae). The parsimony reconciliation rooting approach recovered the terminal branch leading to the *P. tardus* AUREO-like sequence as the minimum duplication-loss root under all 1000 UFBoot trees and under all event-cost schemes (Supplementary Data 7 and 8). The root optimization hence recovered the AUREO-like gene of *P. tardus* as the deepest branching sequence and placed the root outside the AUREO2 and AUREO1a,b,c radiation. A deep branch of the AUREO tree remains weakly resolved and is thus displayed as soft polytomy, reflecting insufficient phylogenetic signal to resolve the exact order of divergence. The combined results of the gene duplication analysis (DLCpar, Treerecs) identified three non-terminal duplication events (i.e., with at least five descendant taxa) across the AUREO gene family, with a re-identification greater than 85% across bootstrapped tree topologies and with a retention of the descendant gene duplicates greater than 20% across descendant taxa. Three of these duplication events occurred before the functional diversification of the AUREOs (see Fig. 2B, red dots), while one occurred within the AUREO 2–Chrysista clade. The duplication events that preceded the functional diversification of the AUREOs indicate that AUREO 2 is the earliest-branching AUREO, separating it from the AUREO 1 clade, with a retention of descendant taxa of 74%. Another duplication event at the base of the AUREO 1 clade led to the separation of AUREO 1a and AUREOs 1b and 1c, with a retention across descendant taxa of 73%. A final duplication event then separates AUREO 1b and 1c. However, both genes of this duplication event remained in 23% across descendant taxa; hence, the retention is much lower than that of the AUREO 2 and AUREO 1a split. Despite possible deviations due to missing transcripts, we overall detected the four AUREOs in a similar total number of species (see Supplementary Data 2), indicating that the four types of AUREOs can be found in most photosynthetic stramenopiles.

### Phylogenetic relationships and domain variability of AUREOs

**Fig. 3:**
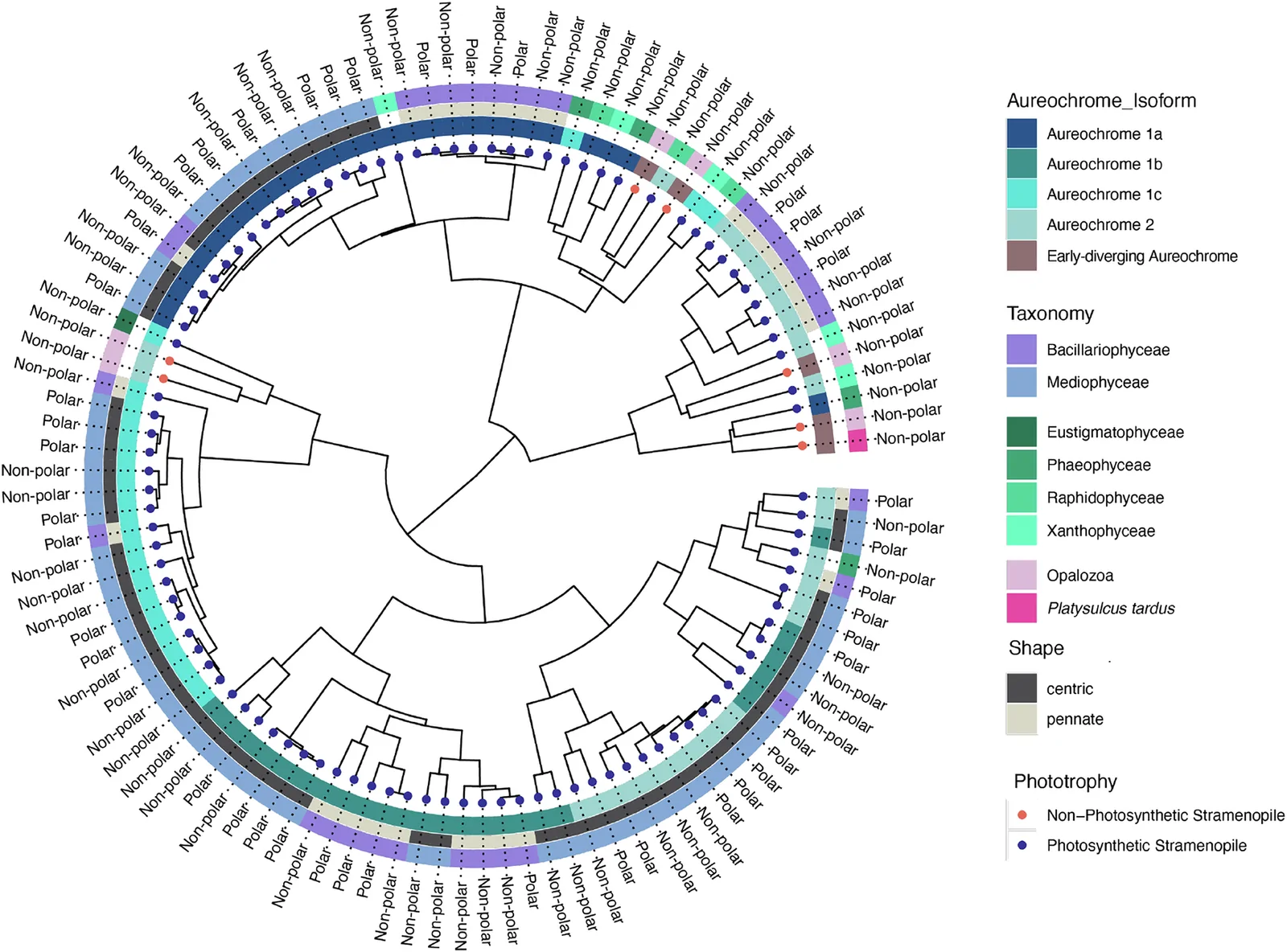
Profileview tree of the LOV-domain of AUREO sequences from transcriptomes and genomes of Southern Ocean and non-polar phytoplankton species. Inner circle dots: Photosynthetic or non-photosynthetic stramenopiles, inner circle: Type of AUREO, middle circle: shape, outer circle: taxonomic group.

In order to compare the LOV-domain of polar and non-polar AUREO sequences more closely, a Profileview-tree was created based exclusively on the LOV-domain of the AUREOs (Supplementary Data 3)^21^. This approach revealed whether the polar AUREO sequences differ from the non-polar ones in terms of the LOV-domain and the associated adaptation to polar light conditions. The AUREO sequences used for the LOV-domain analysis were categorized into the four isotypes based on the full-length sequences. The results show that the LOV-domains of AUREO1a and AUREO1c are more similar to each other than to the other AUREOs (Fig. 3). LOV-domains of AUREO1b and 2 can be found in several clades of the tree. The AUREO2 LOV-domains of centric and pennate diatoms are clearly separated, but there is no separation between LOV-domains from polar and non-polar species (Fig. 3). The higher sequence variability of the AUREO2 LOV-domains is consistent with its separation according to diatom lineage. The separation of AUREO2 LOV-domains in pennate and centric diatoms could indicate a functional difference in the LOV-domains. The LOV-domain of *P. tricornutum* AUREO2 is unable to bind FMN and therefore cannot perceive blue light^8^. Although this observation is currently limited to *P. tricornutum* and *Vaucheria frigida*, it may indicate reduced selective pressure on the AUREO2-LOV domain. Consequently, sequence divergence in this domain may primarily reflect the evolutionary history of the respective species rather than functional constraints associated with blue-light perception. This interpretation is consistent with the observed separation of centric and pennate diatom AUREO2 LOV-domains, suggesting that species phylogeny has a stronger influence on sequence diversification than adaptation of the photoreceptor domain itself.

**Fig. 4:**
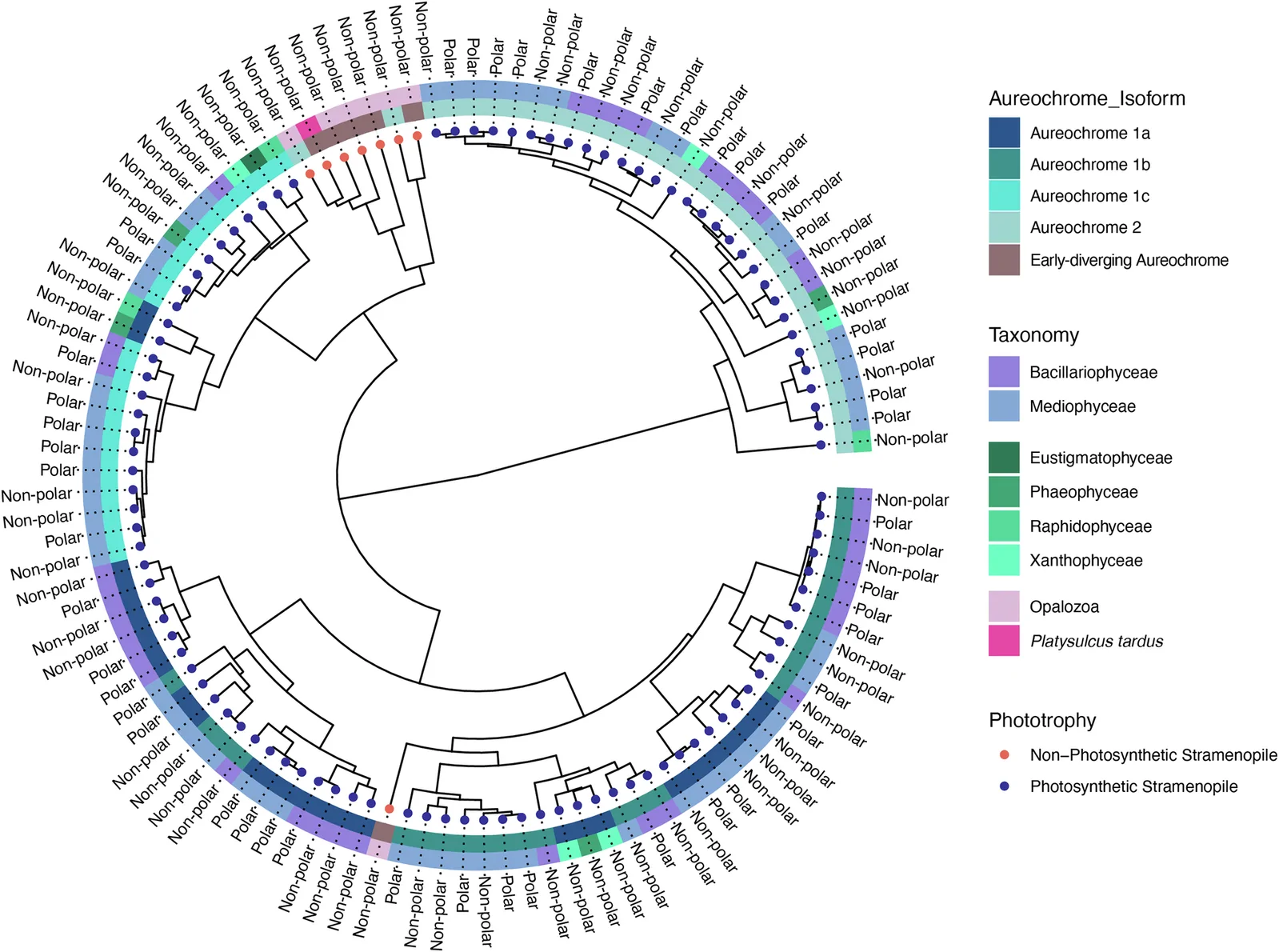
Profileview tree of the bZIP-domain of AUREO sequences from transcriptomes and genomes of phytoplankton species from non-polar and Southern Ocean locations. Inner circle: Photosynthetic or non-photosynthetic stramenopiles, middle circle: type of AUREO, outer circle: taxonomic group.

In a next step, we calculated a further Profileview-tree based on the AUREO bZIP-domains to test whether LOV- and bZIP-domains in the AUREOs show a co-linear evolutionary development^21^. This analysis includes the same sequence dataset as used for the LOV-domain analysis. We found that the bZIP-domains of AUREO 1c and AUREO 2 are more conserved than those of the other AUREOs and that they are located mostly within the same clade (Fig. 4). The bZIP-domain of AUREOs 1a and 1b, instead, are both located in separate clades, indicating a more variable bZIP-domain that may be related to the requirements of specific promoter DNA-binding sites. We could not detect any differentiation between the bZIP-domains of polar and non-polar AUREOs. Thus, AUREO bZIP-domains appear to be conserved with respect to biogeographic distribution, as we detected no clear differentiation between polar and non-polar sequences. However, substantial divergence among AUREO subfamilies was evident, with AUREO1a and AUREO1b forming distinct clades while AUREO1c and AUREO2 remained more closely related (Fig. 4). The bZIP-domains of almost all heterotrophic stramenopile sequences cluster together, indicating that similar regulatory motifs within the promoters are used.

## Discussion

Life in the polar regions is challenged by low temperatures and radically changing light conditions in winter and summer seasons, with permanent light and absolute darkness, respectively. Another obstacle is the low solar angle, which results in a longer passage of photons through the water column, increasing the share of blue light relative to other wavelengths in upper regions, likely increasing its significance as an environmental cue^22^. By integrating global pole-to-pole metatranscriptome with phylogenetic and protein domain-level analysis, we found that high-latitude light conditions are associated with greater LOV-domain protein diversity, while AUREOs form a globally dominant and conserved blue-light receptor family expanding towards early-branching heterotroph stramenopiles.

Our global pole-to-pole metatranscriptome analysis reveals two key patterns. Firstly, the diversity of LOV-domain-containing proteins appears to increase towards high latitudes (Fig. 1A), suggesting that ecological adaptations involving blue-light photoreception could become more important under polar light regimes. Secondly, the AUREOs emerged as the most frequently detected LOV-domain receptor family across all stations (Supplementary Fig. 3), indicating that they play a key role in light sensing in marine stramenopiles.

The higher richness of LOV-domain proteins at polar latitudes aligns with a generally stronger selection for short-wavelength (blue) light-responsive regulation under polar light regimes. Indeed, besides radically changing light conditions in the winter and summer seasons, polar light conditions with a low solar angle require a longer passage of photons through the water column, increasing the share of blue light compared to other wavelengths^23^. The underwater spectral light field also changes depending on ice cover and ice melt, requiring distinct physiological adaptations in polar phytoplankton^24,25^. Also, during the polar winter, lunar illumination has been shown to provide sufficient, deep-penetrating blue light to serve as an environmental cue for meso- and macrozooplankton^26,27^. Recent investigations have shown that the diatom *Fragilariopsis cylindrus* can switch to a hypometabolism during polar winter regimes. In this state, the diatom cells enter a state of quiescence with reduced metabolic and transcriptional activity, during which no cell division occurs^28^. Beyond the unique light environment, temperature itself represents an additional selective force that can shape LOV-domain evolution at higher latitudes. The photochemistry of LOV domains, particularly the kinetics of dark-state reversion following photoactivation, is strongly temperature-dependent, with lower temperatures generally slowing the thermal recovery to the dark state and thereby prolonging the lifetime of the signaling state. Experimental studies have further demonstrated that these recovery kinetics can be evolutionarily tuned across different LOV-containing proteins, indicating that thermal adaptation can directly modify the signaling properties of LOV photoreceptors^29^. Consequently, the low and highly seasonal temperatures characteristic of polar ecosystems may have favored diversification of LOV-domain architectures and photochemical properties, providing a potential explanation for the elevated richness of LOV-domain-containing proteins observed at high latitudes. In sum, adaptation to short-wavelength light, the extreme seasonality of polar light regimes, and potentially the temperature-dependent tuning of LOV photochemistry could have been major drivers of LOV-protein diversification, accounting for the observed increase in receptor architecture diversity toward the poles.

Based on the detection frequency across all stations, we found AUREO proteins with their characteristic combination of an N-terminal bZIP-domain together with a C-terminal LOV-domain to emerge as the most frequently detected LOV-receptor proteins from pole-to-pole (Fig. 1B, Supplementary Fig. 3). Based on the taxonomic transcript annotation, stramenopiles, together with alveolates and haptophytes, are dominating the dataset (Supplementary Fig. 2), possibly explaining the widespread detection of AUREOs. Yet the dominant prevalence of AUREOs relative to other LOV blue-light sensing proteins suggests a conserved and central role in stramenopile photobiology. We are aware that detection frequencies reflect transcript presence, not genomic presence. Hence, the frequencies might be influenced by the community’s temporal and physiological state. The most frequently found domain combinations in LOV-proteins besides AUREOs include a LOV-Histidine-Kinase-HATPase-C-Response-Regulator, WRKY-LOV, and LOV-HSF-DNA-binding (Fig. 1B). These combinations confirm the findings of Coesel et al.^17^. We could also confirm the existence of phototropins (LOV-LOV-pKinase), helmchromes (RGS-LOV-LOV), EF-hand-LOV, Zeitlupe (LOV-Fbox), LOV-Neuralized-LOV, and Homeodomain-LOV motifs in the unicellular protists species of the metatranscriptome datasets, comparable to what is described by Coesel et al.^17^. We further detected previously uncharacterized domain combinations, like LOV-CCT (CONSTANS, CO-like, and TOC1), which could be related to the circadian clock of algae^30^ (Supplementary Fig. 3), C2 + LOV (PF00168, a Ca^2+^-dependant membrane-targeting phospholipid binding domain), dimethylbenzinoamidozole phosphoribosyltransferase (DBI_PRT, PF02277) + LOV, DHBP synthase (PF00926) + LOV, polyketide cyclase (PF03364) + LOV, and SnoaL (PF07366) + LOV, also a polyketide cyclase (Supplementary Fig. 3). Most of these proteins were found only once or a few times in the metatranscriptome dataset. Interestingly, a domain combination of a C-terminal LOV-domain (PF13426) and an N-terminal Polyketide cyclase (PF03364) occurred in 13 distinct metatranscriptome-derived protein sequences. This combination is also found in a predicted protein from the dinoflagellate *Noctiluca scintillans* (accession number A0A7S1FCW1, gene NSCI0253_LOCUS31986, retrieved from InterPro). The specific domain architecture, to our knowledge, has not been functionally characterized or discussed in the literature to date. The dinoflagellate *Noctiluca scintillans* produces a blue bioluminescence with a peak around 470–480 nm^31^, thus this protein could be involved in bioluminescence-dependent secondary metabolite synthesis. The domain combination of an N-terminal DHBP-synthase (PF00926) with a C-terminal LOV-domain (PF13426) was found three times in the metatranscriptome dataset. This previously uncharacterized fusion protein could be involved in secondary metabolite production^32^, for example, in optimizing riboflavin production in response to the daylight cycle. Moreover, a combination of a DHBP synthase followed by a LOV-domain and a GTP cyclohydrolase II (PF00925) could be found in the stramenopile Thraustochytrida (Supplementary Fig. 3). This domain combination also implies a candidate blue-light-regulated riboflavin synthesis with a potential feedback loop, since riboflavin can, besides FMN or FAD, also bind noncovalently to the LOV-domain.

Taxonomically determined LOV-domain sequences from the metatranscriptomic data were analyzed comparatively based on their LOV-domain amino acid sequences. Thereby, the PtAUREO LOV-domains are clustered most closely with the Chlorophyta LOV-domain proteins rather than the LOV-domain of DNA-binding-elements containing proteins of raphidophytes (Fig. 1C). These LOV-domains are phototropin LOV2-domains^33^. A reason for this could be the shared functional constraints for blue-light sensing coupled to transcriptional regulation in PtAUREOs and Chlorophyta LOV-domain proteins, which could drive a rather convergent evolution of the LOV-domain despite taxonomic distance. In phototropins, LOV1 plays a supportive role in stabilizing the protein and regulating its interaction with other molecules, while the conformational change in LOV2 upon light activation enables activation of the kinase domain^34^.

Consistent with this interpretation, our phylogenetic reconstruction indicates an early origin and a broad distribution of AUREOs across lineages, with deep retention and duplication-associated diversification, alongside strong sequence conservation of the key domains.

The phylogenetic analysis of the full-length AUREOs reveals deep, well-supported clade-level duplications, with each major clade containing orthologs from diverse algal lineages, indicating a deep evolutionary origin of AUREOs. The first observed gene duplication separates AUREO1 from AUREO2 proteins, which then further diverge by duplication events into the subclades AUREO 1a and AUREO 1b/AUREO 1c. In addition to these three major duplication events, AUREOs are characterized by further lineage-specific expansions and gene losses within the AUREO clades. Such within-lineage duplications are more recent, occurred after the divergence of the respective taxa, and likely expand blue-light signaling repertoires beyond the conserved AUREO1a/1b/1c/2 duplications. For example, in the xanthophyte *Vaucheria frigida*, two AUREOs have been described originally (VfAUREO1, VfAUREO2)^5^, likely resulting from an early separation of AUREO1 and AUREO2, grouping VfAUREOs as pre-duplication AUREOs. A closer inspection of the *Vaucheria litorea* transcriptome revealed four AUREO sequences (AUREO2, a duplicated AUREO1a and AUREO1c, see Supplementary Data 2). Other lineages, like the benthic diatom *Seminavis robusta*, only harbor 3 genes encoding AUREOs, having apparently lost the gene for AUREO1c (see Supplementary Data 2). In general, gene duplications play a key role in driving functional diversification and specialization^35^. The high retention rates of the duplicated AUREO-genes suggest strong selective advantages, which support the idea that they function as stable regulatory components within stramenopiles rather than short-lived or transient adaptations. The different topology and AUREO grouping of our phylogeny compared to that of Wu et al.^36^. is most likely due to the larger sequence and taxonomic coverage (323 sequences compared to 44 in Wu et al.^36^), and the inclusion of a deep and early-branching stramenopile (*P. tardus*) in our phylogeny and duplication analyses. Rooting on the *P. tardus* branch produces the most parsimonious duplication–loss scenario. This is a common criterion used to root gene-family trees containing paralogues with different ancestral duplications^37–39^. However, we cannot formally exclude the possibility that the last common ancestor of stramenopiles already carried AUREO paralogues, with subsequent lineage-specific losses and paralogue-specific duplications producing a different order of isotype duplication. In line, the unresolved soft polytomy at the deep AUREO tree branches would require additional sampling of early-diverging and heterotrophic stramenopiles to establish whether one or more currently unsampled AUREO-like lineages contribute to this unresolved basal region.

Notably, early-diverging AUREOs were also found in primary heterotrophic stramenopiles, including *P. tardus*, and in non-photosynthetic taxa within the Opalozoa (Fig. 2). We also retrieved early-diverging AUREO sequences from the metatranscriptomes that cluster within the MAST3 lineages (subphylum Opalozoa), underscoring that these heterotrophic AUREO genes are expressed in environmental contexts and serve an ecological function (Supplementary Fig. 5).

The identification of early-diverging AUREO transcripts in non-photosynthetic stramenopiles provides insights into the evolution of AUREOs. As *P. tardus* is a basal lineage of stramenopiles^9,40^, this suggests either retention of an ancestral regulatory gene at the base of stramenopiles or inheritance of a remnant from a photosynthetic ancestor. The latter phenomenon is described for other non-photosynthetic lineages with reduced plastids, like dinotoms^41^. In line with this, our phylogenetic analysis challenges the current assumption that AUREOs are restricted to phototrophs^6^. While stramenopiles most likely originate from a secondary endosymbiosis involving the uptake of a red alga by a eukaryotic cell and its conversion into a plastid^42^, it is not yet resolved whether there is an ancestral AUREO protein in red algae, and whether its first appearance correlates with the acquisition of photosynthetic abilities after secondary endosymbiosis^5,43^. Our metatranscriptome data also include a transcript combining a LOV-domain with a transcription factor domain from red algae (Fig. 1C), which is not a bZIP, but a zinc finger protein (zf_H2C2_5 (PF13909)). The red algal LOV-domain rather clusters with haptophyte-derived LOV/DNA-binding domains, whereas the AUREO LOV-domains cluster with chlorophyte-derived LOV-domains. The presence of AUREOs in non-photosynthetic stramenopiles and their distinction from red-algal blue-light sensing proteins suggest that AUREOs likely evolved within the common ancestor of phototrophic and heterotrophic stramenopiles. Recombinant expression of the *P. tardus* AUREO revealed no visible yellow coloration and no characteristic UV/Vis absorption spectrum indicative of flavin mononucleotide binding, in contrast to PtAUREO1a (Supplementary Figs. 7 and 8). This suggests that the *P. tardus* AUREO does not bind FMN and therefore is unlikely to function as a FMN-dependent blue light photoreceptor. Together, these findings point towards an alternative evolutionary scenario in which canonical FMN-dependent blue-light sensing may not represent an ancestral property of stramenopile AUREOs, but instead could have arisen secondarily within the AUREO1 lineage. In this model, early-diverging AUREOs, such as that from* P. tardus*, may have functioned as light-independent transcriptional regulators, with photoreceptive capacity emerging later through functional specialization of the LOV-FMN module. By contrast, AUREO2 proteins, which can also lack canonical FMN-binding features, may reflect a parallel functional divergence. However, this interpretation does not exclude that photoreceptor function may have been present in the last common ancestor of stramenopiles, followed by multiple losses.

This further broadens the functional role and ecological significance of AUREOs. While AUREO1-class proteins likely function as general light-sensing signal transducers, AUREO2-class proteins, and possibly early-branching AUREOs, as for heterotrophic stramenopiles, may have diversified towards functions beyond direct light sensing. In heterotrophic stramenopiles, these proteins might encompass co-regulation, circadian control, as well as behavioral or metabolic regulation, functions well described, e.g., for fungi^44^ and for heterotrophic protists like *Oxyrrhis marina* in which rhodopsins regulate phototaxis^45^.

Phylogenetic analyses of the 128 amino acid-long LOV-domains reveal that AUREO1a forms the most homogeneous phylogenetic cluster, consistent with its high sequence conservation within its isotypes, regardless of the sampling site. This observation is further supported by pairwise sequence identity analyses, showing that AUREO1a and AUREO1b exhibit the highest similarities within their isotypes (Supplementary Fig. 9). AUREO1c also reveals a relatively stable conservation, while AUREO2 exhibits greater sequence variability within the isotype (Fig. 3, Supplementary Fig. 9). Differences in AUREO2 LOV-domains correlate with diatom morphology and phylogeny, distinguishing pennate from centric diatoms, yet no clustering by origin is observed for any isotype.

AUREO1a and 1b likely experienced selective constraints at the amino acid sequence level to preserve precise blue-light sensing functions: studies in *P. tricornutum* link them to light-independent circadian rhythms, with PtAUREO1a influencing expression patterns of other AUREOs, photosynthetic acclimation, cell cycle regulation, and dimerization of transcription factors^7–10,46^. In contrast, the constitutively expressed AUREO2 in *P. tricornutum* may lack FMN binding for direct photoregulation, thereby reducing selective pressure and permitting greater variability, consistent with a stabilizing or modulatory role^8^.

Recent analyses demonstrate that AUREO bZIPs generally share close structural and functional similarities with other bZIP transcription factors found across plants and animals, exhibiting comparable dimerization properties and DNA-binding affinities for substrate DNA^47^. These conservation patterns are also reflected in the pairwise amino acid identity (Supplementary Fig. 9). While the LOV-domain is more conserved in AUREO1a and AUREO1c, the sequence conservation is lower in AUREO1b and the most variable in AUREO2. Highly conserved bZIP-domains, as in AUREO1b, and 2, may restrict dimer partner binding specificity, while variable bZIP sequences, as in AUREO1a, indicate broader interactions with promoter motifs^48^. This indicates that evolutionary constraints may differ among AUREO isotypes and suggests that the relative conservation of the sensory (LOV) and DNA-binding (bZIP) modules is isotype-specific rather than uniform across the family. The comparatively lower conservation of the AUREO1a bZIP-domain may reflect greater flexibility in DNA interactions or dimerization, consistent with its proposed strong impact on overall gene regulation in *P. tricornutum*^7,49^. The pairwise identity analysis included taxonomically balanced sampling across species within each isotype, ensuring that differences in sequence conservation are not driven by uneven taxonomic distribution.

Our bZIP-domain similarity analysis further revealed that almost all AUREOs (except one) of heterotrophic species form a distinct cluster, suggesting they share a conserved but so far unknown regulatory role distinct from that of phototrophic AUREOs. The clustering of the heterotrophic bZIP domains also further argues against an origin by phototrophic contamination.

The LOV- and bZIP-domain ProfileView trees show a limited concordance (Supplementary Fig. 10), indicating that these domains may have experienced partially independent evolutionary histories, likely due to different evolutionary pressures. One possible evolutionary scenario may involve domain fusion and/or domain shuffling.

Our analysis of the LOV and bZIP-domains revealed no major amino acid changes associated with polar environments, suggesting that AUREOs remain conserved across latitudinal gradients. This is the case for our global analysis as well as a species-to-species comparison of closely related polar diatom species with a non-polar species, which shows no latitudinal effect. The persistence of the same AUREO isotypes from non-polar to polar regions indicates that adaptation to extreme light regimes is not achieved through sequence-level divergence. Instead, our findings support the idea that regulatory tuning, like differential expression patterns, diel oscillations, post-translational modifications, or altered dimerization dynamics, could mediate functional adaptations in polar light regimes. Hence, the conservation of AUREOs across latitudes suggests that the ecological success of these organisms relies more on flexible regulatory mechanisms than on structural diversification of AUREO proteins.

### Conclusion

Overall, our pole-to-pole metatranscriptome analysis reveals a latitudinal increase in the richness of LOV-domain proteins, representing candidate blue-light photoreceptors, suggesting that spectral light sensing by marine protists becomes increasingly important in polar regions. AUREO-type photoreceptors emerge as dominant and consistently detectable blue-light sensors in global phytoplankton communities, with canonical isotypes remaining structurally stable across stramenopiles, consistent with strong selection on both LOV and bZIP-domains and the long-term maintenance of their regulatory functions. The detection of AUREO sequences in early-diverging heterotrophic stramenopiles challenges the view that AUREOs are restricted to phototrophs and instead suggests an earlier origin preceding modern photosynthetic lineages. Crucially, the absence of major sequence differences between polar and non-polar AUREOs suggests that adaptation to extreme polar light conditions is based more on regulatory mechanisms, with the ecological success of polar phytoplankton resulting from a conserved AUREO architecture combined with dynamic regulatory fine-tuning.

## Methods

### Culture conditions and RNA sequencing

The Southern Ocean diatom isolates *Odontella* sp. (isolated in the Atlantic sector of the Southern Ocean during ANT-XXIX/5, 2013), *Thalassiosira* sp. (2019, Station 35, PS117, 69° 04′ S, 17° 19′ W)*, Chaetoceros* sp. (2004, Polarstern EIFEX ANT-XXI/3, 49° 36S, 0° 05E, open waters of Atlantic Southern Ocean), *Pseudo-Nitzschia* sp. (2022, 51° 28′ 41.7′′ S, 49° 11′ 17.8′′ W), and *Fragilariopsis* sp. (isolated from the Weddell Sea during ANT XVI/3 in 1999) were used for this study. All strains were collected outside national jurisdiction (high seas or Antarctic Treaty area) and are therefore not subject to Nagoya Protocol ABS requirements. The strains were cultured in tissue culture flask in Provasoli’s enriched F/2 medium^50^ with 250 mM NaSiO_3_x5H_2_O and 5 mM H_2_SeO_3_ in 34‰ sea salt content. Cells were exposed to a 16:8 light:dark photoperiod at 50 µmol photons m^−2^ s^−1^ and 4 °C. Since some AUREOs follow a diel pattern, RNA samples were taken one hour before, 2 h after, and 6 h after the onset of the light cycle for Long-read RNA Sequencing. For sampling, aliquots of 15 mL were collected from individual cultures and subsequently pooled (total 3 aliquots with a total culture volume of 45 mL for each diatom isolate). Cells were harvested by filtration through polycarbonate membrane filters (Merck KGaA, Darmstadt, Germany) with varying pore sizes based on the cell size of the diatom. *Odontella* sp., *Chaetoceros* sp., and *Thalassiosira* sp. were filtered through 5 µm pore size filters while *Pseudo-Nitzschia* sp. and *Fragilariopsis* sp. were filtered through 2 µm and 1 µm pore sizes, respectively. The cells were rinsed off the filters with 1 mL TriReagent (Sigma-Aldrich, Steinheim, Germany), and RNA isolation was performed as described in detail by Wohlrab et al.^51^.

RNA samples of *Odontella* sp., *Thalassiosira* sp., *Fragilariopsis sp*., and *Pseudo-Nitzschia* sp. were sequenced by PacBio® sequel II long-read isoform sequencing (ISO-seq) at Novogene (Munich, Germany). The RNA sample of *Chaetoceros debilis* was sequenced using Illumina Nextseq 2000 (Illumina, San Diego, CA, USA) using P3 Reagents kit (2 × 150 cycles). The library for Illumina Sequencing was generated using Illumina Stranded mRNA Prep Ligation Kit (Illumina, San Diego, CA, USA) according to the manufacturer’s protocol. Processing of the sequencing data was performed as follows in the ‘Data collection and raw data processing’ as well as the ‘Identification of LOV-domain containing proteins’ sections.

### Data collection and raw data processing

The “Sea of Change” metatranscriptome data collection describes a eukaryotic metatranscriptome project in which samples were taken from the North Pole to the South Pole (“Pole-to-Pole”). The data was obtained from the Joint Genome Institute (JGI) (JGI Proposal ID 532/300780, doi: 10.46936/10.25585/60000951). Protein sequences from genome and transcriptome data were retrieved from the EukProt database (https://evocellbio.com/eukprot/)^52,53^. Additionally, already assembled transcriptome sequences of *Thalassiosira* spp., *Thalassiosira minima*, *Minidiscus* spp., *Minidiscus comicus*, *Minidiscus spinulatus*, and *Minidiscus variabilis* were collected from Guajardo et al.^54^, and of *Chaetoceros debilis* as well as *Fragilariopsis kerguelensis* from Beszteri et al.^55^. Sequences of marine stramenopile species were taken from Labarre et al.^56^, Illumina raw sequences were cleaned with the BBmap (v39.01, bbnorm)^57^ with a target coverage = 200 and a minimum depth = 5, and assembled using Trinity with strand-specific library type RF (--SS_lib_type RF) and no in silico read normalization (--no_normalize_reads)^58^. For ISO-seq long reads, raw reads were cleaned with Lima (v2.9.0)^59^, and refined and clustered with Iso-Seq (v4.0.0^60^). Samtools (v.1.16.1)^61^ was used to convert .bam files into final fasta files. TransDecoder (v5.7.0) was used to determine open reading frames and translations for all nucleotide sequences^62^. Default parameters were used unless otherwise specified above.

### Identification of LOV-domain containing proteins

The presence of LOV-domains in the retrieved sequences was screened using a custom-built hidden Markov model (profile HMMs, http://hmmer.org), built from the published LOV-domain sequence alignment of Coesel et al.^17^. All transcripts that were found to encode a LOV-domain, were further screened for additional domains, including bZIP-domains, by using the PFAM Profile HMM collection and motif-associated significance cut-offs^63^. Sequences containing an N-terminal bZIP-domain followed by a C-terminal LOV-domain were defined as AUREOs.

### Analysis of metatranscriptome data

The taxonomy of transcripts with additional domains besides the LOV-domain was determined with MMseq2^64^ and a custom reference database containing revised sequences of the Marine Microbial Eukaryote Transcriptome Sequencing Project (MMETSP)^65^, from the National Center for Biotechnology Information (NCBI), and from the MarRef databases hosted at the Marine Metagenomics Portal (https://sfb.mmp2.sigma2.no/) (for details, see Supplemental Data 5). Sequences assigned as microeukaryotes were kept for the analysis of the diversity of LOV-domain encoding transcripts. To test whether LOV-domain diversity is latitudinally structured independently of background taxonomic diversity and sequencing depth, we fitted a Generalized Additive Model (GAM, with a negative binomial error distribution, suitable for count data) with LOV-domain richness as the response variable with the R package mgcv and used residual diagnostics to validate model assumptions^66^. Based on our hypothesis that biogeographic gradients may influence blue-light sensory diversity, we modeled latitude using a smooth spline to capture potential nonlinear patterns. As LOV-domain richness may be related to the overall taxonomic complexity of the community, we included the Shannon Index (order-level taxonomic diversity) as a linear covariate. The respective order-level clade counts were created from the MMseq2 taxonomy report files with Pavian^67^. Sequencing depth (gigabases of each sample) was included as another covariate. This enabled us to determine the influence of latitude on LOV-domain diversity while accounting for community diversity and sequencing effort.

### Phylogenetic reconstruction and duplication history of AUREOs

We reconstructed the evolutionary history of AUREOs across the stramenopiles by identifying their taxonomic distributions, inferring their phylogenetic relationships, and approximating gene duplication events using reconciliation models. Specifically, we screened all stramenopile proteomes from the EukProt database v3^52^ for AUREO candidates as described above. We inferred the species phylogeny for all species with AUREOs using OrthoFinder v3.1^68^ with default settings, using the recommended MSA-based workflow with FAMSA alignments, FastTree gene-tree inference, and MCL clustering. The topology of the obtained species tree was compared to the published reference phylogeny by Cho et al.^69^, and the tree was rooted accordingly. The gathered AUREO sequences were aligned using MAFFT v7.490 with the –auto option and multithreading enabled and a high-accuracy iterative refinement approach (L-INS-i)^70^. Poorly aligned, gap-rich regions were trimmed from the alignment using trimAI v1.5^71^ with the -gappyout option. To account for potential heterogeneity in evolutionary rates across AUREO domains (bZIP, linker sequence, LOV), we defined alignment-based domain partitions for subsequent phylogenetic inference. Partitioned maximum-likelihood phylogenetic inference was performed in IQ-TREE2 v2.3.6 using ModelFinder with partition merging enabled, with branch support estimated from 1000 ultrafast bootstrap replicates and 1000 SH-aLRT replicates^72^. As there are no AUREOs outside the stramenopiles, there are no AUREO homologs available as an external outgroup to root the AUREO gene tree. Therefore, we evaluated the AUREO root using species-tree-informed duplication-loss reconciliation^38,73^. Root optimization was performed using Treerecs v1.2^73^, with 1000 UFBoot trees inferred from IQ-TREE2 (see above), as well as an independently inferred stramenopile species tree^69^. As duplication and loss costs are free parameters, we repeated the root optimization process under several event-cost schemes to confirm that the inferred root is insensitive to event weighting. Root optimization was performed under the following schemes: equal duplication and loss costs (D = 1, L = 1); a strongly duplication-weighted scheme (D = 1000, L = 1); and intermediate weightings (D = 2 and D = 10, L = 1). Hence, in congruence with the species tree, the AUREO tree was rooted using the *Platysulcus tardus* AUREO sequence. Gene duplication histories were reconstructed with DLCpar v2.0.1 (dlcpar_search option with default settings) in order to obtain primary annotations and a locus-specific reconciled gene tree^74^. Retention scores were then calculated for each duplication event, representing the proportion of descendant species that retained paralogs in both post-duplication lineages. To evaluate the robustness of each duplication event with respect to phylogenetic uncertainty, we additionally reconciled every IQ-TREE-derived ultrafast bootstrap gene tree using Treerecs v1.2^73^ with default reconciliation settings, and assessed the robustness of each duplication event by determining how often an equivalent duplication was recovered across the reconciled bootstrap trees. This provided a direct estimate of duplication support under phylogenetic uncertainty. Environmental AUREO sequences of likely heterotrophic stramenopile origin were incorporated by adding them to the trimmed AUREO reference alignment with MAFFT v7.490 with the –add and –keeplength options, preserving alignment length and column structure. The added sequences were then placed on the prior established AUREO reference tree topology with IQ-TREE2 v2.3.6 (see above). The AUREO reference tree was thereby used as a fixed backbone, and the same domain partitions, model-selection settings, support metrics and outgroup were applied as described above. All trees and duplication annotations were visualized either with FigTree v1.4.4 (https://github.com/rambaut/figtree) or with R v4.4.3 using the treeio, ggtree, ggplot2 and ape packages^75–78^.

### Domain-specific phylogenies of LOV-domain-containing proteins including AUREOs

In order to identify fine-scale motif variation that could reveal functional differences and local adaptations to polar conditions within the LOV and bZIP domains, we constructed trees that represent sequence space clusters with Profileview^21^. We used this approach once for all LOV-domains with taxonomic annotations retrieved from the metatranscriptomes but excluded AUREOs due to the high number of sequences. Instead, we added reference AUREOs from *Phaetodactylum tricornutum* (Joint Genome Protein Identifiers 49116, 49458, 56742, and 56060)^79^. This served to identify the similarity of the blue-light sensing motif. In a second approach, we included AUREOs from our sequencing approaches, i.e., representing Southern Ocean species and specifically closely related non-polar species, with further representative AUREO sequences of distinct stramenopile lineages. This approach served to identify likely local adaptations to polar light regimes in AUREOs as well as to resolve a general functional clustering of the AUREO isoforms across different lineages. Therefore, AUREO sequences were retrieved from transcriptomic data of public databases (EukProt), and data from our own sequencing approaches were filtered for the blue-light sensing motif with hidden Markov models^52^. For the LOV and bZIP-domains, separate trees were constructed using Profileview^21^. To obtain Profileview-trees of the LOV and the bZIP-domain of sub selected AUREOs, two domain model library were built using Pfam domain libraries for the PAS (PF00989.29), PAS_2 (PF08446.15), PAS_3 (PF08447.16), PAS_4 (PF08448.14), PAS_5 (PF07310.17), PAS_6 (PF08348.15), PAS_7 (PF12860.11), PAS_8 (PF13188.11), PAS_9 (PF13426.11), PAS10 (PF13596.10), PAS_11 (PF14598.10), PAS_12 (PF18095.5), GdpP_PAS (PF21370.1), PdeA_PAS (PF21815.1), and bZIP_1 (PF00170.25), bZIP_2 (PF07716.19), bZIP_C (PF12498.12), and bZIP_Maf (PF03131.21) domain, respectively. For sequences containing more than one LOV-domain (metatranscriptome data), single LOV-domains were extracted from the hmm-search result with an additional 25 amino acids as flanking regions. All trees were visualized in R v4.4.1 using the ggtree, ggtreeExtra, ggnewscale, ggplot2, tidyverse, and ape packages^75–77,80–82^.

## Supplementary information

Supplementary Information

Description of Additional Supplementary Files

Data Set 1

Data Set 2

Data Set 3

Data Set 4

Data Set 5

Data Set 6

Data Set 7

Data Set 8

## Data Availability

The datasets generated in this study are stored and publicly available in the repositories described below. Transcriptome data generated within this study are available in Supplementary Data 4 and publicly available at Figshare.com (https://doi.org/10.6084/m9.figshare.33394543)^83^. Additional publicly available metatranscriptome data that support the findings of this study are available in the Joint Genome Institute with the Proposal ID 532/300780 (doi: 10.46936/10.25585/60000951)^84^, as well as genome and transcriptome data from EUKPROT V3^52,53^, https://doi.org/10.6084/m9.figshare.21586065.v1 (2022), from the National Center for Biotechnology Information, accession number PRJNA706094^54^, from the European Nucleotide Archive (ENA) at the European Molecular Biological Laboratory – European Bioinformatics Institute (EMBI_EBI) accession number PRJEB18576, and from Figshare under the project number https://doi.org/10.6084/m9.figshare.c.5008046^56^. The source data for Fig. 1 is provided in Supplementary Data 1, for Fig. 2 in Supplementary Data 2, and for Figs. 3 and 4 in Supplementary Data 3. All relevant data are also available upon request from the authors. The corresponding author will be responsible for requests for access to data.

## Code availability

All analyses were performed with previously published pipelines and scripts as stated in the “Methods” section. Software versions and analysis parameters are described in the “Methods”.

## References

[1] Häfker, N. S. et al. Rhythms and clocks in marine organisms. *Annu. Rev. Mar. Sci.* **15**, 509–538, 10.1146/annurev-marine-030422-113038 (2023). 36028229

[2] Valle, K. C. et al. System responses to equal doses of photosynthetically usable radiation of blue, green, and red light in the marine diatom *Phaeodactylum tricornutum*. *PLoS ONE* **9**, e114211, 10.1371/journal.pone.0114211 (2014). PMC4254936 25470731

[3] Jaubert, M., Bouly, J.-P., d’Alcalà, M. R. & Falciatore, A. Light sensing and responses in marine microalgae. *Curr. Opin. Plant Biol.* **37**, 70–77, 10.1016/j.pbi.2017.03.005 (2017). 28456112

[4] Duanmu, D., Rockwell, N. C. & Lagarias, J. C. Algal light sensing and photoacclimation in aquatic environments. *Plant Cell Environ.* **40**, 2558–2570, 10.1111/pce.12943 (2017). PMC5705019 28245058

[5] Takahashi, F. et al. AUREOCHROME, a photoreceptor required for photomorphogenesis in stramenopiles. *Proc. Natl. Acad. Sci. USA* **104**, 19625–19630, 10.1073/pnas.0707692104 (2007). PMC2148339 18003911

[6] Kroth, P. G., Wilhelm, C. & Kottke, T. An update on aureochromes: phylogeny–mechanism–function. *J. Plant Physiol.* **217**, 20–26, 10.1016/j.jplph.2017.06.010 (2017). 28797596

[7] Mann, M. et al. The aureochrome photoreceptor PtAUREO1a is a highly effective blue light switch in diatoms. *Iscience* **23**, 10.1016/j.isci.2020.101730 (2020). PMC7670200 33235981

[8] Banerjee, A. et al. Allosteric communication between DNA-binding and light-responsive domains of diatom class I aureochromes. *Nucleic Acids Res.* **44**, 5957–5970, 10.1093/nar/gkw420 (2016). PMC4937327 27179025

[9] Jirsová, D. & Wideman, J. G. Integrated overview of stramenopile ecology, taxonomy, and heterotrophic origin. *ISME J*. **18**. 10.1093/ismejo/wrae150 (2024). PMC11412368 39077993

[10] Schellenberger Costa, B. et al. Blue light is essential for high light acclimation and photoprotection in the diatom *Phaeodactylum tricornutum*. *J. Exp. Bot.* **64**, 483–493, 10.1093/jxb/ers340 (2013). PMC3542041 23183259

[11] Madhuri, S., Lepetit, B., Fürst, A. H. & Kroth, P. G. A knockout of the photoreceptor PtAureo1a results in altered diel expression of diatom clock components. *Plants* **13**. 10.3390/plants13111465 (2024). PMC11174801 38891274

[12] Costa, B. S. et al. Correction: Aureochrome 1a is involved in the photoacclimation of the diatom *Phaeodactylum tricornutum*. *PLoS ONE* **8**. 10.1371/annotation/7000208e-7505-4c2d-beed-fc99236bbe9f (2013). PMC3792145 24116244

[13] Rockwell, N. C., Su, Y. S. & Lagarias, J. C. Phytochrome structure and signaling mechanisms. *Annu. Rev. Plant Biol.* **57**, 837–858, 10.1146/annurev.arplant.56.032604.144208 (2006). PMC2664748 16669784

[14] Fortunato, A. E. et al. Diatom phytochromes reveal the existence of far-red-light-based sensing in the Ocean. *Plant Cell* **28**, 616–628, 10.1105/tpc.15.00928 (2016). PMC4826011 26941092

[15] DeOliveira, C. C. & Crane, B. R. A structural decryption of cryptochromes. *Front. Chem.* **12**, 1436322. 10.3389/fchem.2024.1436322 (2024). PMC11362059 39220829

[16] Hallmann, A. Sensing a rainbow of colors: algal photoreceptors. *Front. Plant Sci.* **16**, 1684559 (2025). 10.3389/fpls.2025.1684559. PMC12558739 41164245

[17] Coesel, S. N. et al. Diel transcriptional oscillations of light-sensitive regulatory elements in open-ocean eukaryotic plankton communities. *Proc. Natl. Acad. Sci. USA* **118**, e2011038118. 10.1073/pnas.2011038118 (2021). PMC8017926 33547239

[18] Poliner, E. et al. Aureochromes maintain polyunsaturated fatty acid content in *Nannochloropsis oceanica*. *Plant Physiol.* **189**, 906–921, 10.1093/plphys/kiac052 (2022). PMC9157131 35166829

[19] Saikkonen, K. et al. Toward an integrated understanding of how extreme polar light regimes, hybridization, and light-sensitive microbes shape global biodiversity. *One Earth* **7**, 1529–1541, 10.1016/j.oneear.2024.08.002 (2024).

[20] Gilbertson, R., Langan, E. & Mock, T. Diatoms and their microbiomes in complex and changing polar oceans. *Front. Microbiol*. **13**. 10.3389/fmicb.2022.786764 (2022). PMC8991070 35401494

[21] Vicedomini, R., Bouly, J. P., Laine, E., Falciatore, A. & Carbone, A. Multiple profile models extract features from protein sequence data and resolve functional diversity of very different protein families. *Mol. Biol. Evol*. **39**. 10.1093/molbev/msac070 (2022). PMC9016551 35353898

[22] Ragni, M. & Ribera D’Alcalà, M. Light as an information carrier underwater. *J. Plankton Res.* **26**, 433–443, 10.1093/plankt/fbh044 (2004).

[23] Connan-McGinty, S. et al. Midnight sun to polar night: a model of seasonal light in the Barents Sea. *J. Adv. Model. Earth Syst.* **14**, e2022MS003198. 10.1029/2022MS003198 (2022).

[24] Soja-Woźniak, M. et al. Loss of sea ice alters light spectra for aquatic photosynthesis. *Nat. Commun.* **16**, 4059. 10.1038/s41467-025-59386-x (2025). PMC12043827 40307241

[25] Hoppe, C. J. M. et al. Photosynthetic light requirement near the theoretical minimum detected in Arctic microalgae. *Nat. Commun.* **15**, 7385. 10.1038/s41467-024-51636-8 (2024). PMC11375000 39231958

[26] Shen, A. et al. Altered underwater light characteristics impact photoreceptive mesozooplankton and macrozooplankton from physiological responses to community dynamics. *Ecol. Indic.* **178**, 114011. 10.1016/j.ecolind.2025.114011 (2025).

[27] Last, K. S., Hobbs, L., Berge, J., Brierley, A. S. & Cottier, F. Moonlight drives ocean-scale mass vertical migration of zooplankton during the Arctic winter. *Curr. Biol.* **26**, 244–251, 10.1016/j.cub.2015.11.038 (2016). 26774785

[28] Joli, N. et al. Hypometabolism to survive the long polar night and subsequent successful return to light in the diatom *Fragilariopsis cylindrus*. *N. Phytol.* **241**, 2193–2208, 10.1111/nph.19387 (2024). 38095198

[29] Zoltowski, B. D., Vaccaro, B. & Crane, B. R. Mechanism-based tuning of a LOV domain photoreceptor. *Nat. Chem. Biol.* **5**, 827–834, 10.1038/nchembio.210 (2009). PMC2865183 19718042

[30] Liu, H., Zhou, X., Li, Q., Wang, L. & Xing, Y. CCT domain-containing genes in cereal crops: flowering time and beyond. *Theor. Appl. Genet.* **133**, 1385–1396, 10.1007/s00122-020-03554-8 (2020). 32006055

[31] Letendre, F., Blackburn, A. & Twardowski, M. Linking peak intensity of mechanically stimulated bioluminescence and cell surface area in dinoflagellates. *Biol. Open* **14**. 10.1242/bio.062190 (2025). PMC12486207 40856025

[32] Sasso, S., Pohnert, G., Lohr, M., Mittag, M. & Hertweck, C. Microalgae in the postgenomic era: a blooming reservoir for new natural products. *FEMS Microbiol. Rev.* **36**, 761–785, 10.1111/j.1574-6976.2011.00304.x (2012). 22091538

[33] Freddolino, L., Dittrich, M. & Schulten, K. Dynamic switching mechanisms in LOV1 and LOV2 domains of plant phototropins. *Biophys. J.* **91**, 3630–3639, 10.1529/biophysj.106.088609 (2006). PMC1630464 16935961

[34] Hart, J. E. & Gardner, K. H. Lighting the way: Recent insights into the structure and regulation of phototropin blue light receptors. *J. Biol. Chem.* **296**, 100594. 10.1016/j.jbc.2021.100594 (2021). PMC8086140 33781746

[35] Birchler, J. A. & Yang, H. The multiple fates of gene duplications: Deletion, hypofunctionalization, subfunctionalization, neofunctionalization, dosage balance constraints, and neutral variation. *Plant Cell* **34**, 2466–2474, 10.1093/plcell/koac076 (2022). PMC9252495 35253876

[36] Wu, Y. et al. Genome-wide identification and analysis of the aureochrome gene family in *Saccharina japonica* and a comparative analysis with six other algae. *Plants* **11**, 2088, 10.3390/plants11162088 (2022). PMC9416419 36015392

[37] Górecki, P. & Eulenstein, O. Algorithms: simultaneous error-correction and rooting for gene tree reconciliation and the gene duplication problem. *BMC Bioinforma.* **13**, S14. 10.1186/1471-2105-13-S10-S14 (2012). PMC3382441 22759419

[38] Szöllősi, G. J., Rosikiewicz, W., Boussau, B., Tannier, E. & Daubin, V. Efficient exploration of the space of reconciled gene trees. *Syst. Biol.* **62**, 901–912, 10.1093/sysbio/syt054 (2013). PMC3797637 23925510

[39] Chen, K., Durand, D. & Farach-Colton, M. NOTUNG: a program for dating gene duplications and optimizing gene family trees. *J. Comput Biol.* **7**, 429–447, 10.1089/106652700750050871 (2000). 11108472

[40] Shiratori, T., Nakayama, T. & Ishida, K. -i A new deep-branching stramenopile, *Platysulcus tardus* gen. nov., sp. nov. *Protist* **166**, 337–348, 10.1016/j.protis.2015.05.001 (2015). 26070192

[41] Hehenberger, E., Imanian, B., Burki, F. & Keeling, P. J. Evidence for the retention of two evolutionary distinct plastids in dinoflagellates with diatom endosymbionts. *Genome Biol. Evol.* **6**, 2321–2334, 10.1093/gbe/evu182 (2014). PMC4217693 25172904

[42] Sibbald, S. J. & Archibald, J. M. Genomic insights into plastid evolution. *Genome Biol. Evol.* **12**, 978–990, 10.1093/gbe/evaa096 (2020). PMC7348690 32402068

[43] Pietluch, F., Mackiewicz, P., Ludwig, K. & Gagat, P. A new model and dating for the evolution of complex plastids of red alga origin. *Genome Biol. Evol*. **16**. 10.1093/gbe/evae192 (2024). PMC11413572 39240751

[44] Corrochano, L. M., Corrochano-Luque, M., Franco-Cano, A., Gutiérrez, G. & Cánovas, D. Light sensing in fungi. *Curr. Biol.* **35**, R1134–R1138, 10.1016/j.cub.2025.10.041 (2025). 41330315

[45] Hartz, A. J., Sherr, B. F. & Sherr, E. B. Photoresponse in the heterotrophic marine dinoflagellate *Oxyrrhis marina*. *J. Eukaryot. Microbiol.* **58**, 171–177, 10.1111/j.1550-7408.2011.00529.x (2011). 21332875

[46] Manzotti, A. et al. Circadian regulation of key physiological processes by the RITMO1 clock protein in the marine diatom *Phaeodactylum tricornutum*. *N. Phytol.* **246**, 1724–1739, 10.1111/nph.70099 (2025). PMC12018780 40172009

[47] Khamaru, M., Bose, D., Deb, A. & Mitra, D. Decoding sequence-structure-function-evolution of basic leucine zippers of aureochromes from heterokont algae. *J. Struct. Biol.* **218**, 108283. 10.1016/j.jsb.2025.108283 (2026). 41468981

[48] Nijhawan, A., Jain, M., Tyagi, A. K. & Khurana, J. P. Genomic survey and gene expression analysis of the basic leucine zipper transcription factor family in rice. *Plant Physiol.* **146**, 323–324, 10.1104/pp.107.112821 (2007). PMC2245831 18065552

[49] Im, S. H. et al. Identification of promoter targets by Aureochrome 1a in the diatom *Phaeodactylum tricornutum*. *J. Exp. Bot.* **75**, 1834–1851, 10.1093/jxb/erad478 (2023). PMC10967249 38066674

[50] Smith, W. L. & Chanley, M. H. *Culture of Marine Invertebrate Animals* (Springer, 1975).

[51] Wohlrab, S., Selander, E. & John, U. Predator cues reduce intraspecific trait variability in a marine dinoflagellate. *BMC Ecol.* **17**, 8. 10.1186/s12898-017-0119-y (2017). PMC5327569 28241803

[52] Richter, D. J. et al. EukProt: a database of genome-scale predicted proteins across the diversity of eukaryotes. Peer Community Journal, 2: e56, https://peercommunityjournal.org/item/10_24072_pcjournal_173/ (2022).

[53] Poh, Y.-P. EukProt v3: a database of genome-scale predicted proteins across the diversity of eukaryotes. figshare. https://figshare.com/articles/dataset/TCS_tar_gz/21586065 (2022).

[54] Guajardo, M., Groussman, R. D. & Vault, D. Transcriptomes from the diatoms *Thalassiosira* and *Minidiscus* from the English Channel and Antarctica. Zenodo. https://daniel-vaulot.fr/files/papers/Guajardo_2021_SciData.pdf (2021).

[55] Beszteri, S., Thoms, S., Benes, V., Harms, L. & Trimborn, S. The response of three southern ocean phytoplankton species to ocean acidification and light availability: a transcriptomic study. *Protist* **169**, 958–975, 10.1016/j.protis.2018.08.003 (2018). 30453274

[56] Labarre, A. et al. Comparative genomics reveals new functional insights in uncultured MAST species. *ISME J.* **15**, 1767–1781, 10.1038/s41396-020-00885-8 (2021). PMC8163842 33452482

[57] Bushnell, B. *BBMap: A Fast, Accurate, Splice-Aware Aligner*. https://www.osti.gov/biblio/1241166 (Ernest Orlando Lawrence Berkeley National Laboratory, 2014).

[58] Grabherr, M. G. et al. Full-length transcriptome assembly from RNA-Seq data without a reference genome. *Nat. Biotechnol.* **29**, 644–652, 10.1038/nbt.1883 (2011). PMC3571712 21572440

[59] Pacific, B. *Lima: Demultiplex Barcoded PacBio Samples*. https://github.com/PacificBiosciences/barcoding (Pacific Biosciences, 2017).

[60] Pacific, B. *IsoSeq: Scalable De Novo Isoform Discovery from Long-read PacBio Sequencing Data*. https://github.com/PacificBiosciences/IsoSeq (Pacific Biosciences, 2018).

[61] Li, H. et al. The sequence alignment/map format and SAMtools. *Bioinformatics* **25**, 2078–2079, 10.1093/bioinformatics/btp352 (2009). PMC2723002 19505943

[62] Haas, B. J. *TransDecoder: Identify Candidate Coding Regions within Transcript Sequences*. https://github.com/TransDecoder/TransDecoder (TransDecoder project Broad Institute, 2013).

[63] Mistry, J. et al. Pfam: The protein families database in 2021. *Nucleic Acids Res.* **49**, D412–D419, 10.1093/nar/gkaa913 (2021). PMC7779014 33125078

[64] Mirdita, M., Steinegger, M., Breitwieser, F., Söding, J. & Levy Karin, E. Fast and sensitive taxonomic assignment to metagenomic contigs. *Bioinformatics* **37**, 3029–3031, 10.1093/bioinformatics/btab184 (2021). PMC8479651 33734313

[65] Van Vlierberghe, M., Di Franco, A., Philippe, H. & Baurain, D. Decontamination, pooling and dereplication of the 678 samples of the Marine Microbial Eukaryote Transcriptome Sequencing Project. *BMC Res. Notes* **14**, 306. 10.1186/s13104-021-05717-2 (2021). PMC8353744 34372933

[66] Wood, S. N. Fast stable restricted maximum likelihood and marginal likelihood estimation of semiparametric generalized linear models. *J. R. Stat. Soc. B* **73**, 3–36, 10.1111/j.1467-9868.2010.00749.x (2010).

[67] Breitwieser, F. P. & Salzberg, S. L. Pavian: interactive analysis of metagenomics data for microbiome studies and pathogen identification. *Bioinformatics* **36**, 1303–1304, 10.1093/bioinformatics/btz715 (2019). PMC8215911 31553437

[68] Emms, D., Liu, Y., Belcher, L., Holmes, J. & Kelly, S. OrthoFinder: scalable phylogenetic orthology inference for comparative genomics. *bioRxiv* 10.1101/2025.07.15.664860 (2025).

[69] Cho, A., Lax, G. & Keeling, P. J. Phylogenomic analyses of ochrophytes (stramenopiles) with an emphasis on neglected lineages. *Mol. Phylogen. Evol.* **198**, 108120. 10.1016/j.ympev.2024.108120 (2024). 38852907

[70] Katoh, K. & Standley, D. M. MAFFT multiple sequence alignment software version 7: improvements in performance and usability. *Mol. Biol. Evol.* **30**, 772–780, 10.1093/molbev/mst010 (2013). PMC3603318 23329690

[71] Capella-Gutiérrez, S., Silla-Martínez, J. M. & Gabaldón, T. trimAl: a tool for automated alignment trimming in large-scale phylogenetic analyses. *Bioinformatics* **25**, 1972–1973, 10.1093/bioinformatics/btp348 (2009). PMC2712344 19505945

[72] Minh, B. Q. et al. IQ-TREE 2: new models and efficient methods for phylogenetic inference in the genomic era. *Mol. Biol. Evol.* **37**, 1530–1534, 10.1093/molbev/msaa015 (2020). PMC7182206 32011700

[73] Comte, N. et al. Treerecs: an integrated phylogenetic tool, from sequences to reconciliations. *Bioinformatics* **36**, 4822–4824, 10.1093/bioinformatics/btaa615 (2020). 33085745

[74] Mawhorter, R., Liu, N., Libeskind-Hadas, R. & Wu, Y.-C. Inferring Pareto-optimal reconciliations across multiple event costs under the duplication-loss-coalescence model. *BMC Bioinforma.* **20**, 639. 10.1186/s12859-019-3206-6 (2019). PMC6916210 31842732

[75] Paradis, E., Claude, J. & Strimmer, K. APE: analyses of phylogenetics and evolution in R language. *Bioinformatics* **20**, 289–290, 10.1093/bioinformatics/btg412 (2004). 14734327

[76] Wickham, H. *ggplot2: Elegant Graphics for Data Analysis*. https://ggplot2.tidyverse.org (Springer, 2016).

[77] Yu, G., Smith, D. K., Zhu, H., Guan, Y. & Lam, T. T.-Y. ggtree: an R package for visualization and annotation of phylogenetic trees with their covariates and other associated data. *Methods Ecol. Evol.* **8**, 28–36, 10.1111/2041-210X.12628 (2017).

[78] Wang, L.-G. et al. Treeio: an R package for phylogenetic tree input and output with richly annotated and associated data. *Mol. Biol. Evol.* **37**, 599–603, 10.1093/molbev/msz240 (2019). PMC6993851 31633786

[79] Bowler, C. et al. The Phaeodactylum genome reveals the evolutionary history of diatom genomes. *Nature* **456**, 239–244, 10.1038/nature07410 (2008). 18923393

[80] Wickham, H. et al. Welcome to the tidyverse. J*. Open Source Softw.* **4**, 1686. 10.21105/joss.01686 (2019).

[81] Xu, S. et al. ggtreeExtra: compact visualization of richly annotated phylogenetic data. *Mol. Biol. Evol.* **38**, 4039–4042, 10.1093/molbev/msab166 (2021). PMC8382893 34097064

[82] Campitelli, E. ggnewscale: Multiple fill and colour scales in ‘ggplot2’. *Zenodo*. https://CRAN.R-project.org/package=ggnewscale (2024).

[83] Misir, M. K., Peter, G. & Wohlrab, S. Pole-to-pole LOV-domain receptor diversity points to an early stramenopile origin and global dominance of Aureochromes - Supplementary Data 4. 10.6084/m9.figshare.33394543.v1 (2026). PMC13737086 42982881

[84] Mock, T. Metatranscriptome of marine eukaryotic phytoplankton communities from South Atlantic Ocean. 10.46936/10.25585/60000951 (2018).

