# Supplementary material for: Pole-to-pole LOV-domain receptor diversity points to an early stramenopile origin and global dominance of aureochromes
Source: Commun Biol. 2026 Oct 31;9:1464. doi: 10.1038/s42003-026-11067-1 (PMC13737086; doi:10.1038/s42003-026-11067-1)
Supplement: Supplementary file 1 [file 42003_2026_11067_MOESM1_ESM.pdf]

Supplementary Information

Pole-to-pole LOV-domain receptor diversity points to an early stramenopile origin and global dominance of Aureochromes

Supplementary Figure 1: Sample sites used for the metatranscriptomic analysis across the globe based on the ‘Sea of Change: Eukaryotic Phytoplankton Communities in the Arctic Ocean’ dataset. 2
Supplementary Figure 2: Supplementary Figure 2: Relative taxonomic composition of eukaryotic transcripts per station inferred from the ‘Sea of Change’ metatranscriptome dataset, sorted by latitude. 3
Supplementary Figure 3: Relative abundance of different LOV-domain proteins in combination with other domains across the different stations and areas across the globe, inferred from transcripts of the ‘Sea of Change ‘metatranscriptomic dataset. 4
Supplementary Figure 4: Schematic visualization of unique eukaryotic domain combinations identified in the ‘Sea of Change’ metatranscriptome dataset. 5
Supplementary Figure 5: Phylogenetic placement of AUREO sequences from heterotrophic MAST species identified within the Sea of Change metatranscriptome dataset. 6
Supplementary Method: Recombinant expression of AUREO proteins, quantification, and characterization 7
Supplementary Figure 6: Verification of *P. tardus* early branching AUREO purification via Ni-NTA affinity chromatography 8
Supplementary Figure 7: Extracted recombinant proteins of PtAUREO1a (left) and *P. tardus* AUREO (right) (30 µM concentration of each protein) without any addition of additional flavin mononucleotide (FMN) 9
Supplementary Figure 8: UV/Vis spectra of full-length recombinant PtAUREO1a and *P. tardus* early branching AUREO. 9
Supplementary Method: Pairwise amino acid identity analysis 10
Supplementary Figure 9: Pairwise amino acid identity of AUREO isotypes. 10
Supplementary Method: Co-phylogeny analysis of LOV and bZIP domains 11
Supplementary Figure 10: Co-phylogenetic comparison of LOV and bZIP domain trees. 11

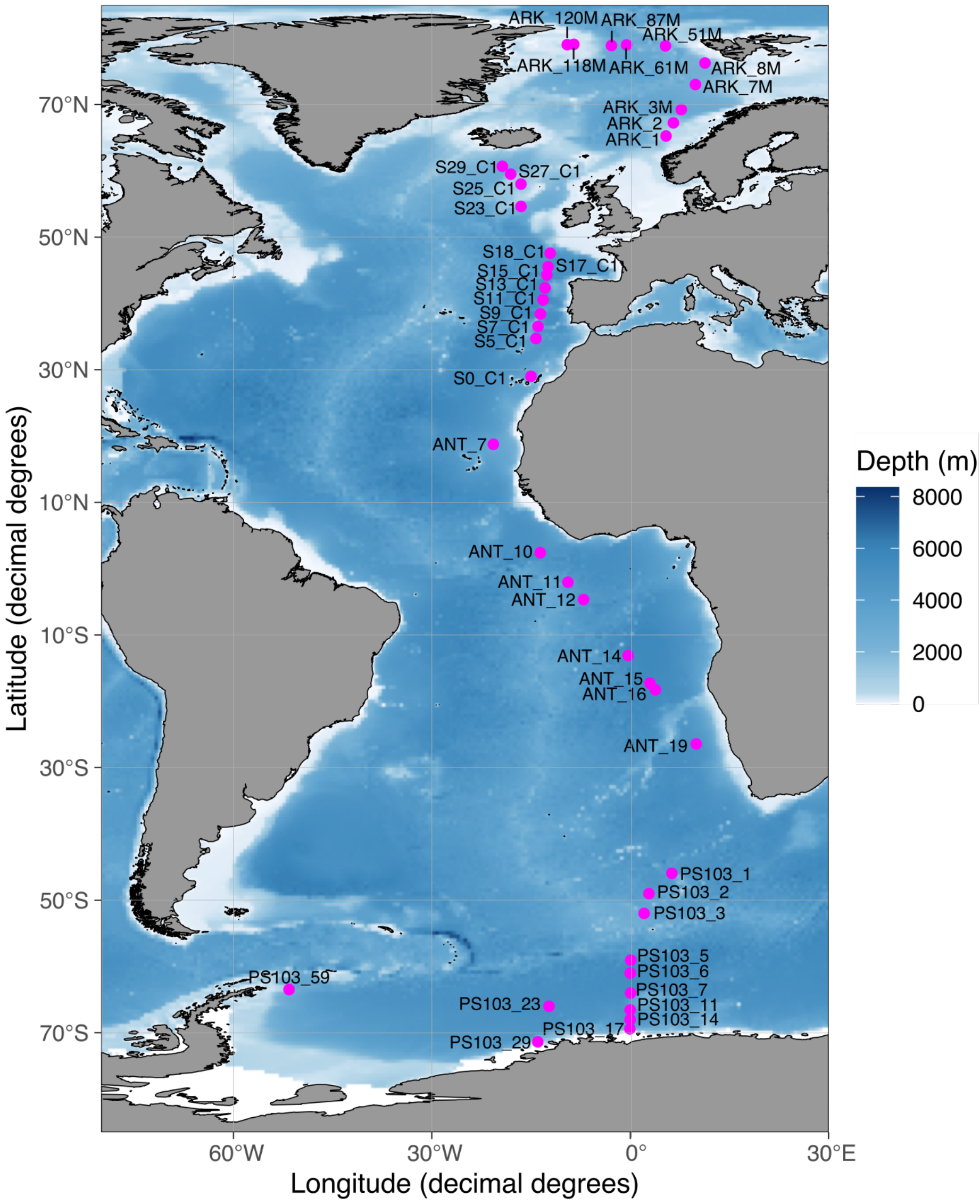

Supplementary Figure 1: Sample sites used for metatranscriptomic analysis across the globe based on the 'Sea of Change' dataset. Magenta-colored dots show the geographic position of each station, while the label indicates the station code. The figure was created in RStudio using ggplot2 and ggOceanMaps using the coordinates of station points from the 'Sea of Change' dataset. The basemap and bathymetry were provided through ggOceanMaps [1-3].

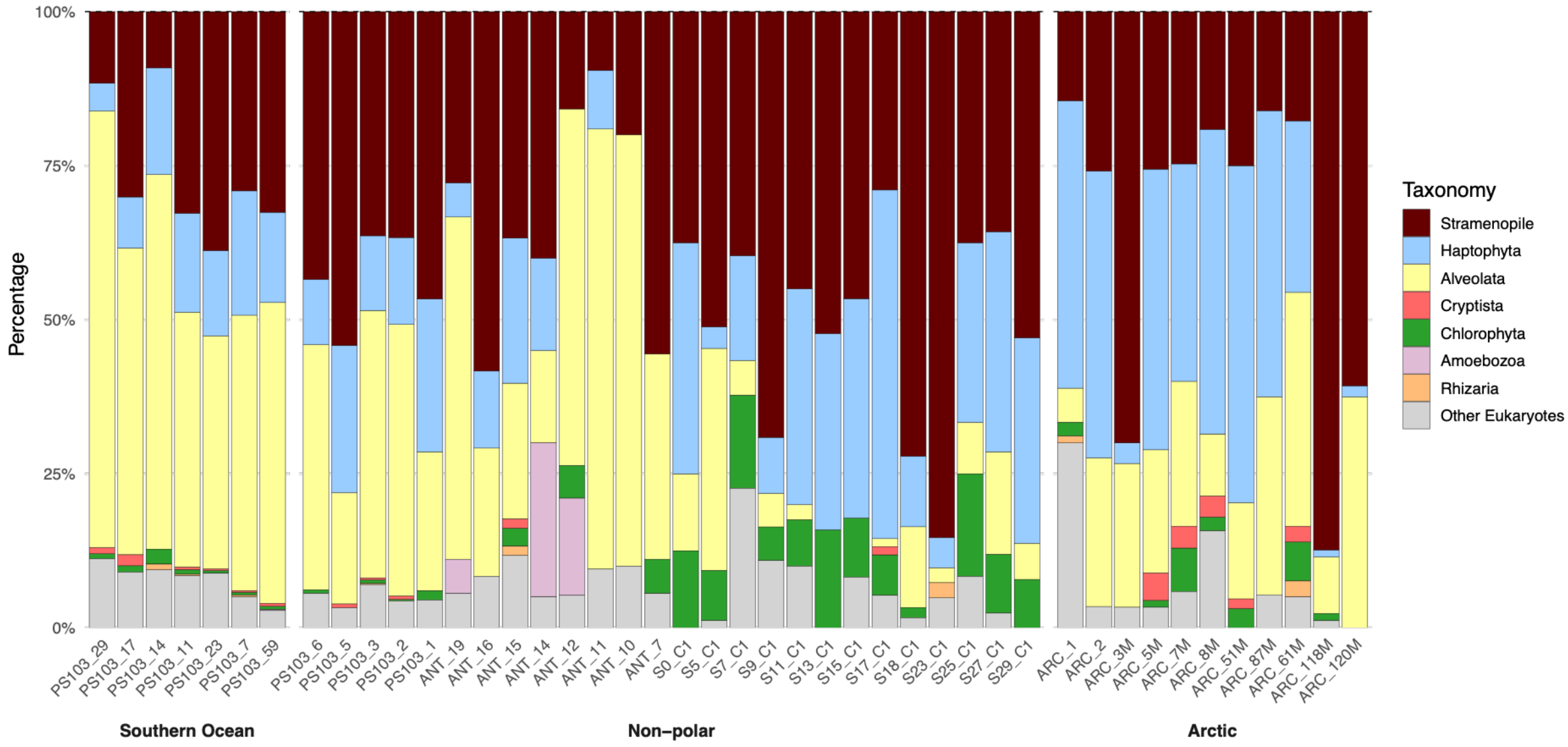

Supplementary Figure 2: Relative taxonomic composition of eukaryotic transcripts per station inferred from the ‘Sea of Change’ metatranscriptome dataset, sorted by latitude. Taxa without reliable classification were grouped as *Other Eukaryotes* and bacterial sequences were removed prior to analysis. Colors indicate major taxonomic categories.

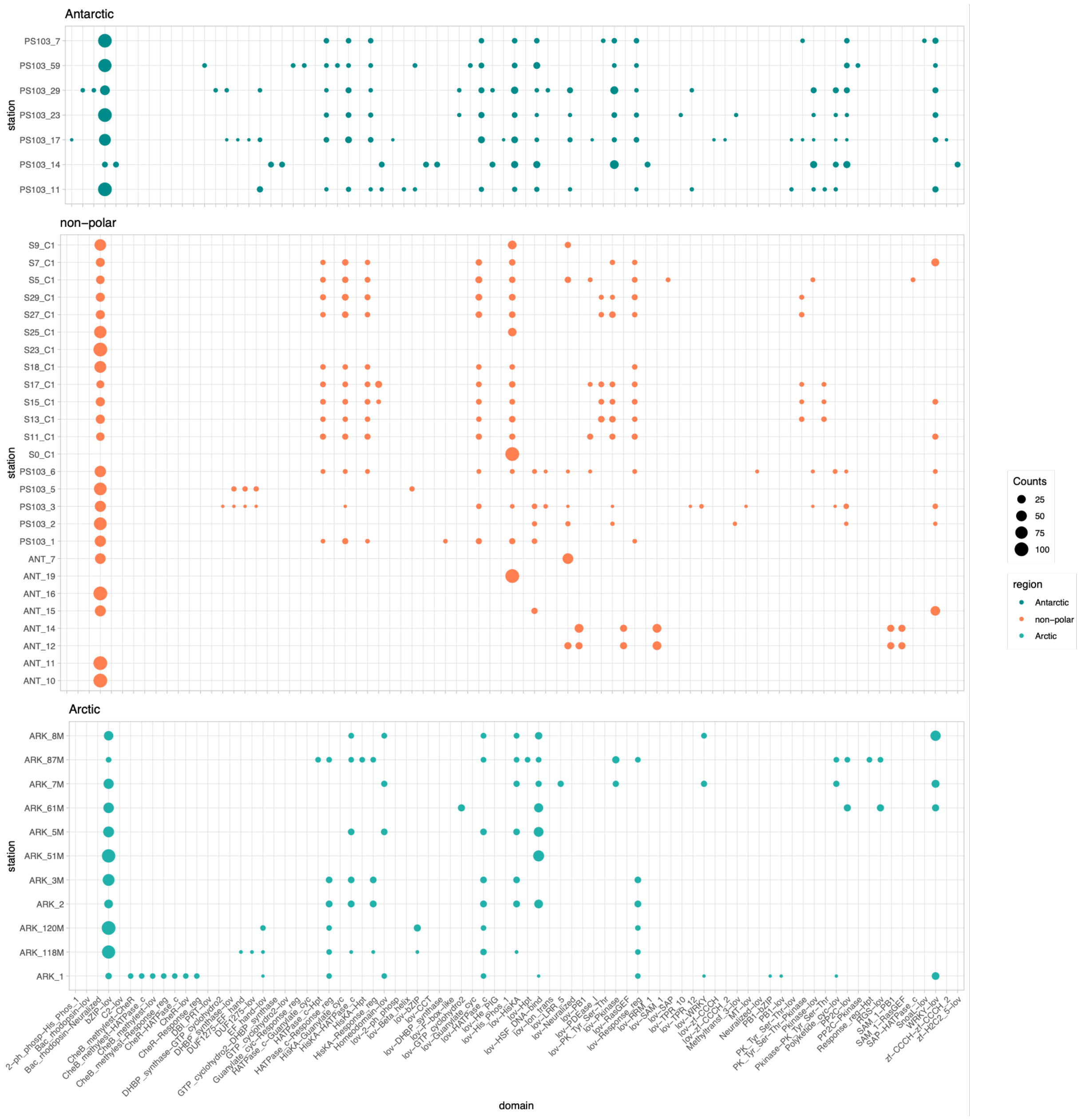

Supplementary Figure 3: Relative abundance of different eukaryotic LOV-domain proteins in combination with other domains across the different stations and areas across the globe, inferred from transcripts of the ‘Sea of Change’ metatranscriptomic dataset. Stations are ordered by latitude and grouped into three regions: Antarctic (cyan), non-polar (orange), and Arctic (light blue). Each dot represents the proportional contribution (%) of a specific domain to the total domain count of a station. Dot size corresponds to the relative percentage within each station.

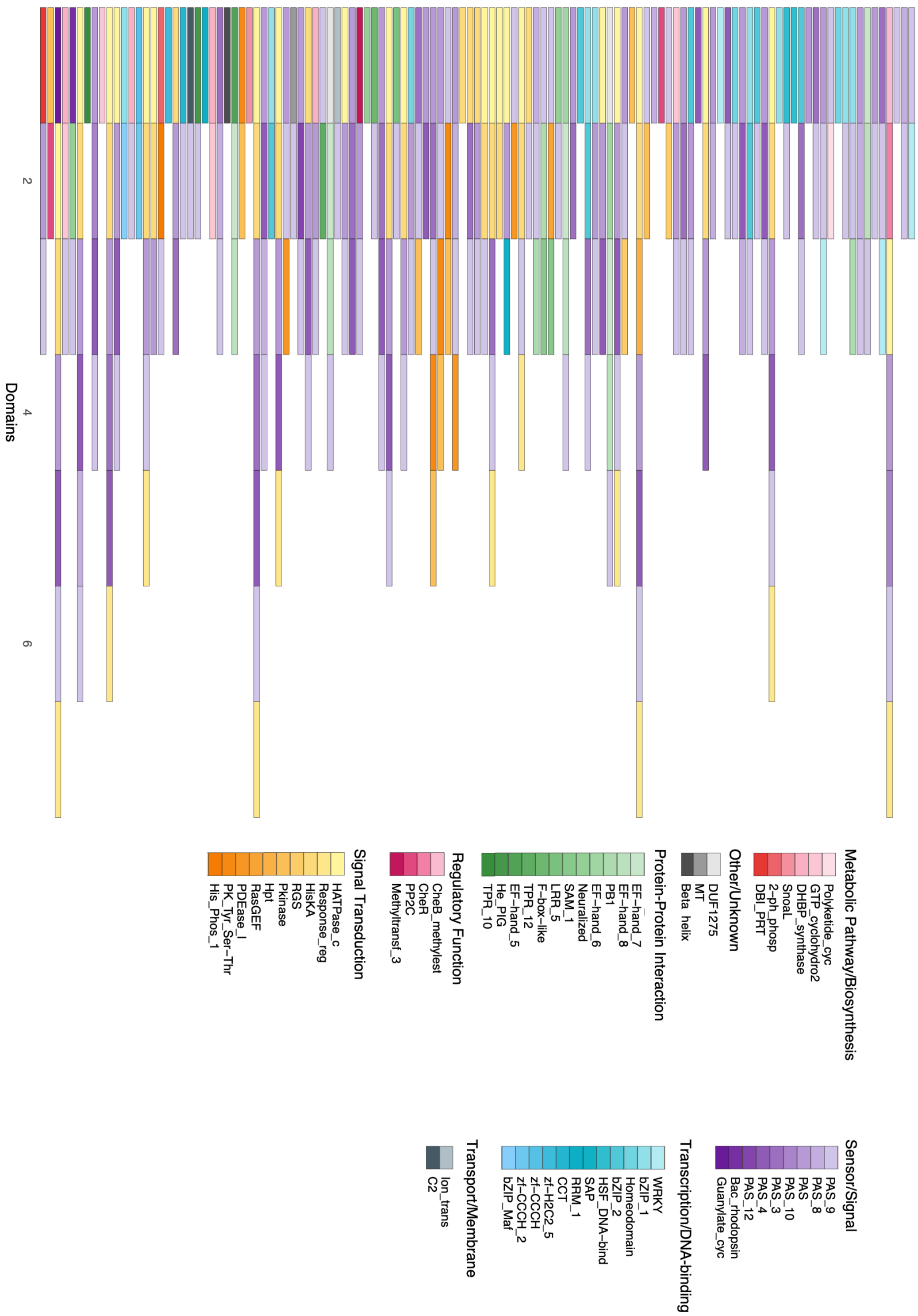

Supplementary Figure 4: Schematic visualization of unique eukaryotic domain combinations identified in the 'Sea of Change' metatranscriptome dataset. Each row represents a unique domain architecture. Domains were categorized into functional groups as shown in the legend.

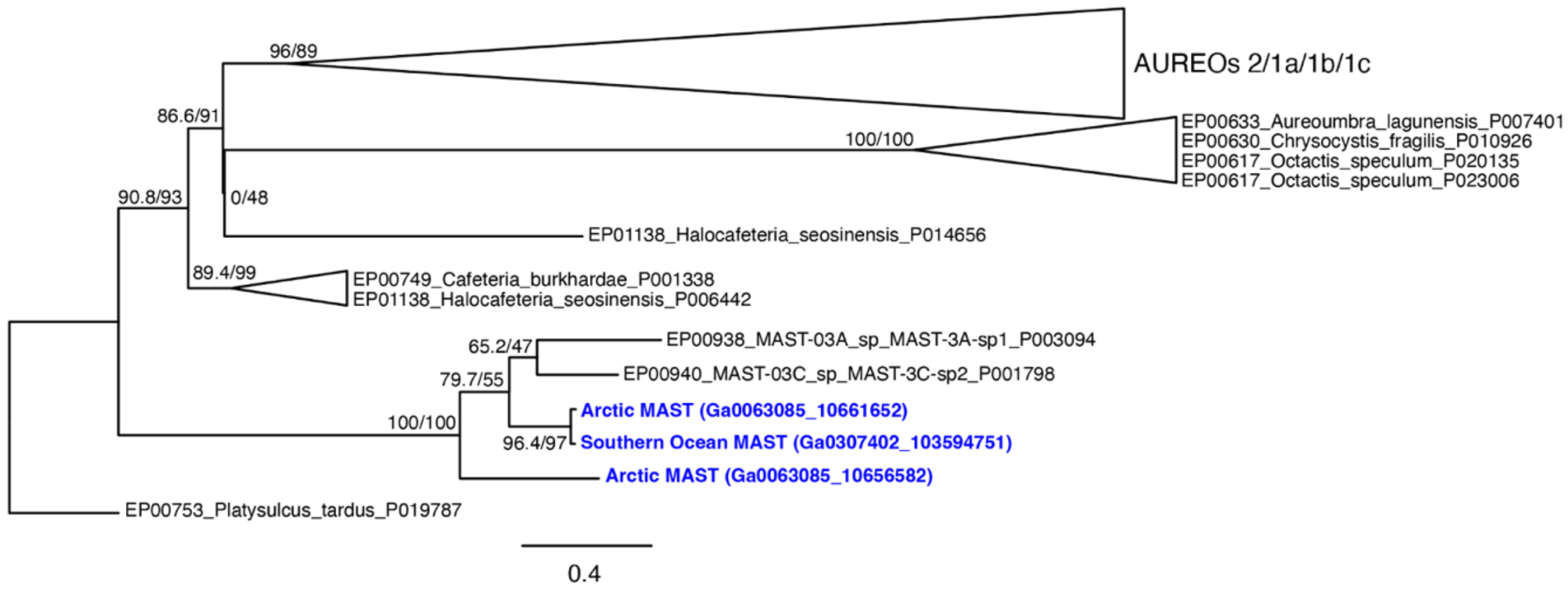

Supplemental Figure 5: Phylogenetic placement of AUREO sequences from heterotrophic MAST species identified within the Sea of Change metatranscriptome dataset.

Supplementary Note 1: Recombinant expression of AUREO proteins, quantification, and characterization

The full-length *P. tardus* AUREO gene, together with an N-terminal His6-fusion, was synthesized by BioCat GmbH (Heidelberg, Germany) and inserted into a pET-28a(+) vector. The *P. tricornutum* AUREO1a (PtAUREO1a) was amplified from cDNA and inserted into a pET-28(+) vector by Im et al. (2024). The respective vectors were transformed into *E. coli* Rosetta (DE3) cells (Merck Millipore, Darmstadt, Germany) for the expression of recombinant proteins, with Kanamycin as a selective antibiotic.

E. coli transformants were checked via colony PCR using Taq-Polymerase S (Genaxxon, Ulm, Germany) and primers binding to the pET28a(+) vector (Forward: GGGGAATTGTGAGCGGATAACA, Reverse: CCAACTCAGCTTCCTTTCGG). An overnight culture was transferred to a volume of 2 L of LB-medium (with 50 µg/mL Kanamycin). Induction occurred after the cells reached an OD600 of 0.5-0.6 by adding IPTG to a final concentration of 0.5 mM. [PK1] Cells were grown overnight at 16°C on a rotating plate (220 rpm) and harvested by centrifugation for 20 minutes at 4°C and 4200 rpm. Cells were resuspended in lysis buffer (pH 8, 50 mM Tris-HCl, 300 mM NaCl, 10 mM Imidazole, 5% glycerol) and incubated on ice with adage of a final concentration of 0.3 mg/mL lysozyme and 1 mM PMSF[PK2] . Cells were disrupted by sonication, and soluble proteins were separated by centrifugation (45 minutes at 4°C, 20.000 rpm).

Ni-NTA columns (Macherey-Nagel, Düren, Germany) [PK3] were equilibrated with lysis buffer, then the lysate was applied and washed 3x with wash buffer (pH 8, 50mM Tris-HCl, 300 mM NaCl, 25 mM Imidazole). Elution occurred with elution buffer (pH 8, 50 mM Tris-HCl, 300 mM NaCl, 250 mM Imidazole) after a 5-minute incubation. Protein concentration was determined using Pierce™ BCA Protein Assay Kit (Thermo Scientific™, Karlsruhe, Germany), and elution fractions with the highest concentrations were combined. The protein was concentrated, and the buffer was exchanged using Amicon® Ultra centrifugal filters with a 10 kDa MWCO (Sigma-Aldrich Chemie GmbH, Taufkirchen, Germany). Buffer exchange, including removal of imidazole, was performed with 5 wash steps using protein buffer (pH 8, 50 mM Tris-HCl, 50 mM NaCl). The concentration was determined, and the protein concentration was set to 30 µM.

For the Flavin mononucleotide (FMN) treatment, proteins were supplemented with a 4-fold molar excess of FMN (Merck KGaA, Darmstadt, Germany). Samples were then incubated for 2 hours at 4°C in the dark. Non-bound FMN was removed using Amicon® Ultra centrifugal filters with a 10 kDa MWCO (Sigma-Aldrich Chemie GmbH, Taufkirchen, Germany). UV/Vis spectra of the samples were measured using a JASCO UV-Vis Spectrophotometer V-630 (JASCO Deutschland GmbH, Pfungstadt, Germany). Spectra were measured from 340 nm to 500 nm, with or without additional FMN treatment. For samples treated with FMN, a protein buffer treated with FMN in the same way as the sample was used as a reference. For non-FMN-treated samples, pure protein buffer was used as a reference.

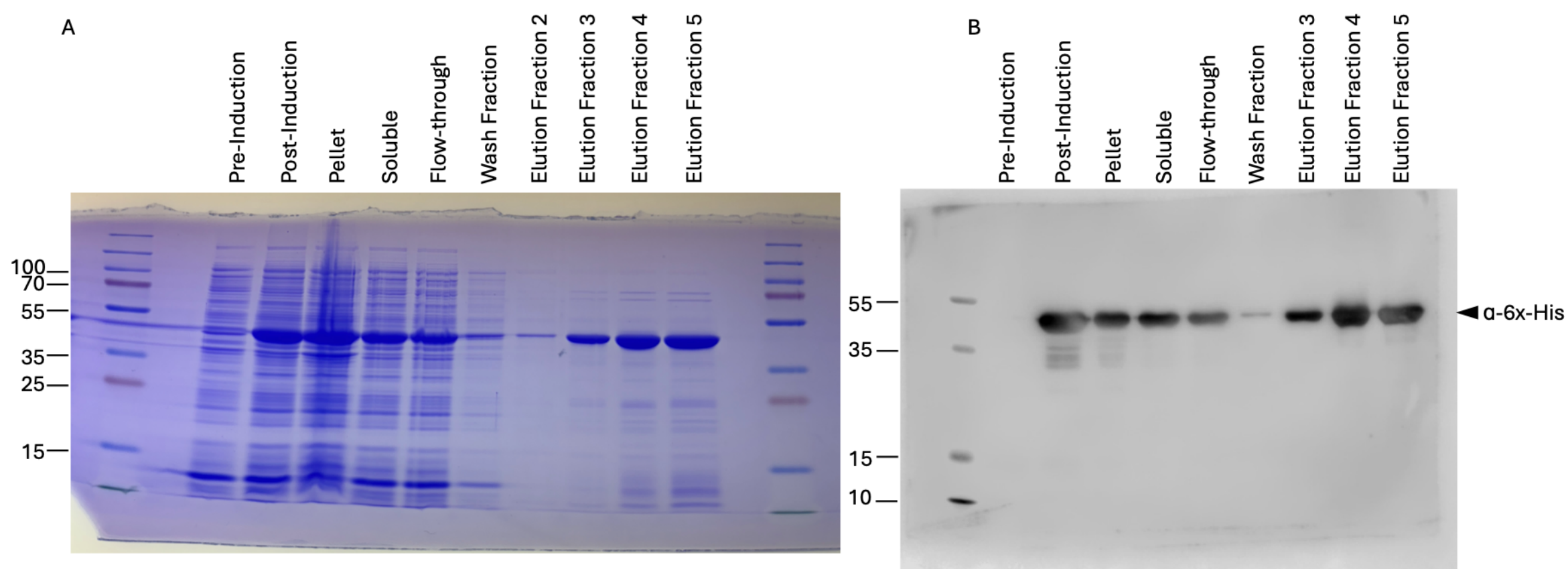

Supplementary Figure 6: Verification of P. tardus early AUREO purification via Ni-NTA affinity chromatography. A) Coomassie Brilliant Blue R-250 staining of proteins before and after protein purification. B) Immunoblot of proteins before and after purification. Proteins were separated by SDS-PAGE and analysed with 6x-His Tag antibody. The approximate molecular weight of the *P. tardus* early-branching AUREO with a 6x-His Tag is 43.4 kDa. The gel and immunoblot images are shown as uncropped images.

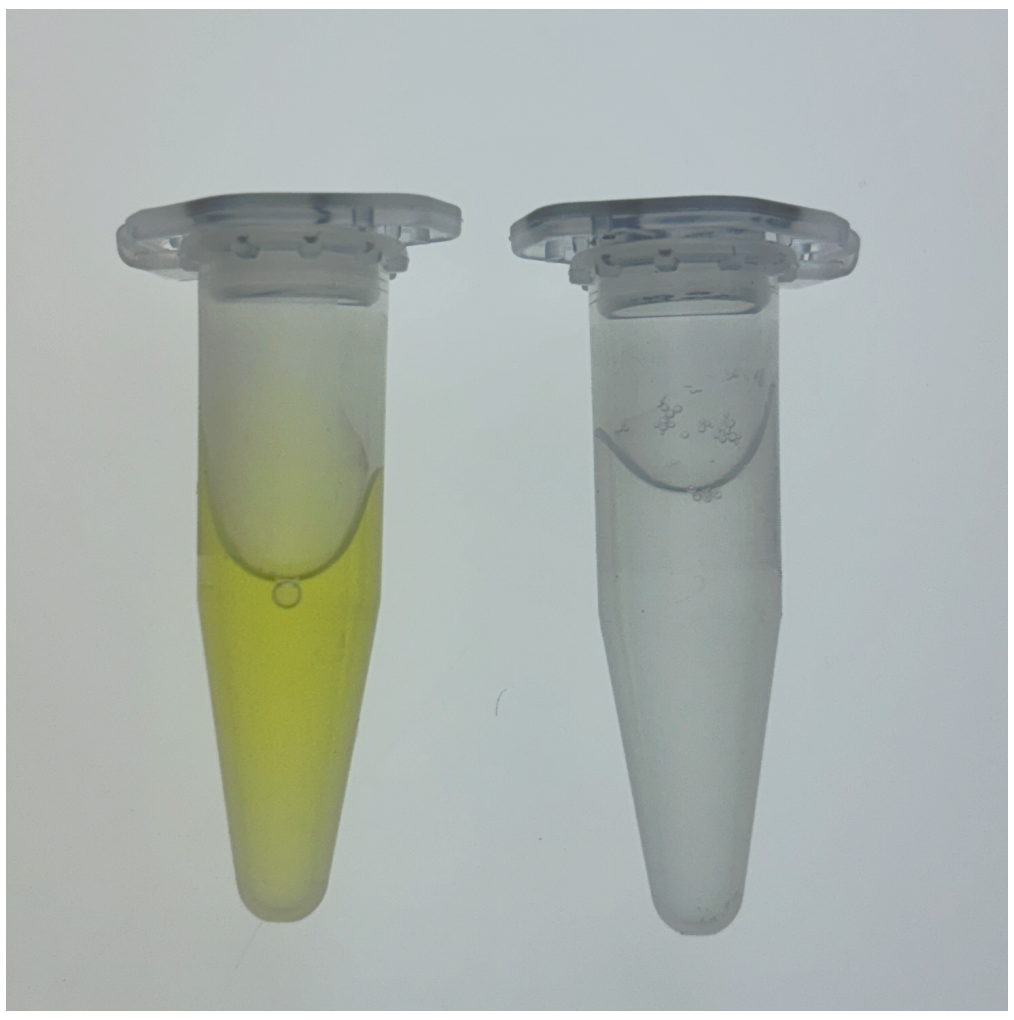

Supplementary Figure 7: Extracted recombinant proteins of PtAUREO1a (left) and P. tardus AUREO (right) (30 µM concentration of each protein) without any addition of additional flavin mononucleotide (FMN).

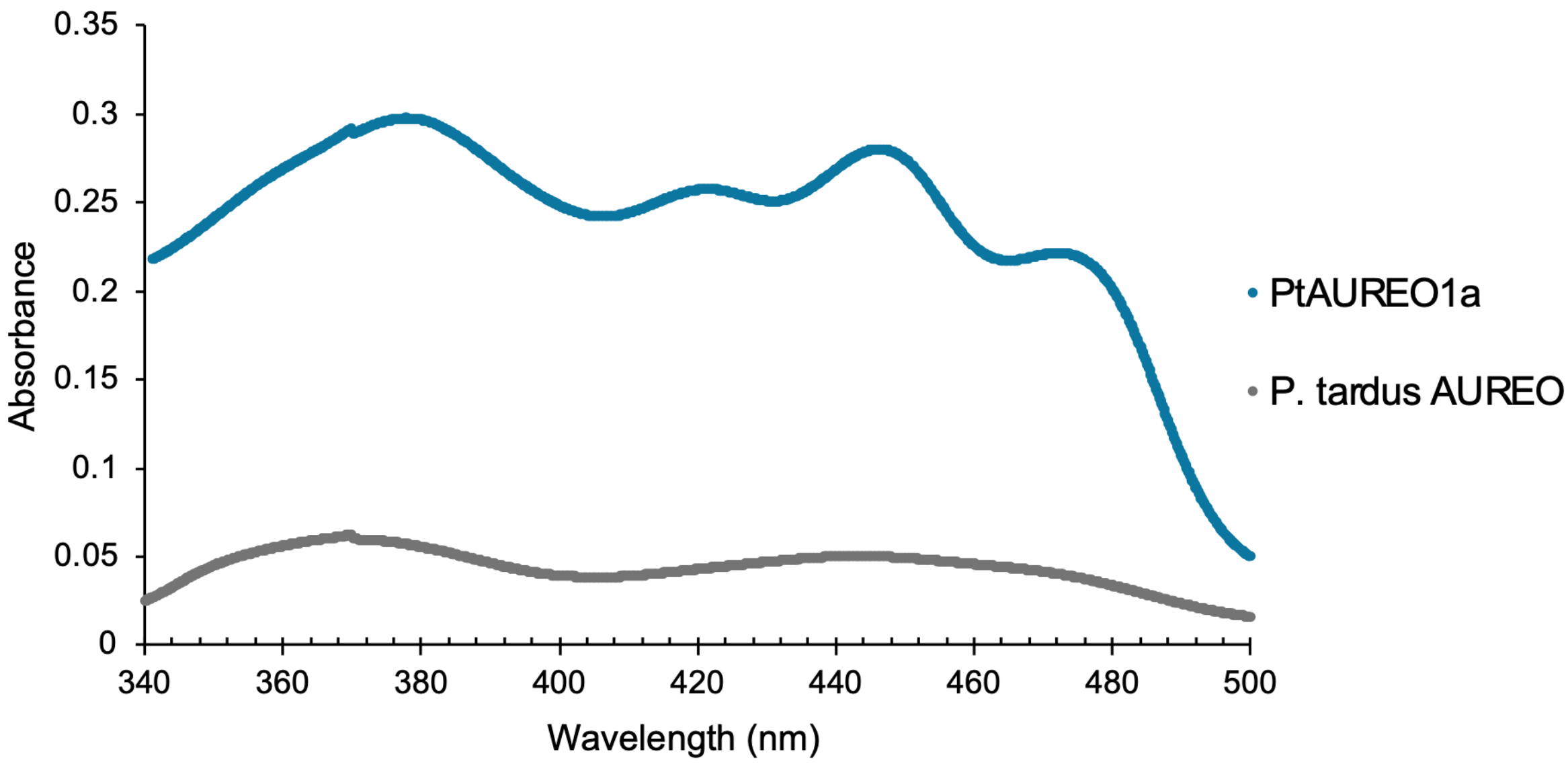

Supplementary Figure 8: UV/Vis spectra of full-length recombinant PtAUREO1a and *P. tardus* early branching AUREO. Proteins were set to a molarity of 30 µM.

Supplementary Note 2: Pairwise amino acid identity analysis

To perform a pairwise amino acid identity analysis, multiple sequence alignments of both the LOV and the bZIP domain from each AUREO isotype (AUREO1a, 1b, 1c, and 2) were imported in FASTA format and processed in R v4.4.3 using the seqinr package [4]. For each alignment, all unique sequence pairs were compared. Pairwise identity was defined as the proportion of identical amino acids across aligned positions, excluding sites where both sequences contained gap characters ("-"). Positions containing gaps in only one of the sequences were included in the calculation. Pairwise identity values are expressed as percentages. Pairwise identity values were used to compute the mean and standard deviation per group. Statistical comparisons between LOV and bZIP domains within each isotope were performed using two-sided Wilcoxon rank-sum tests.

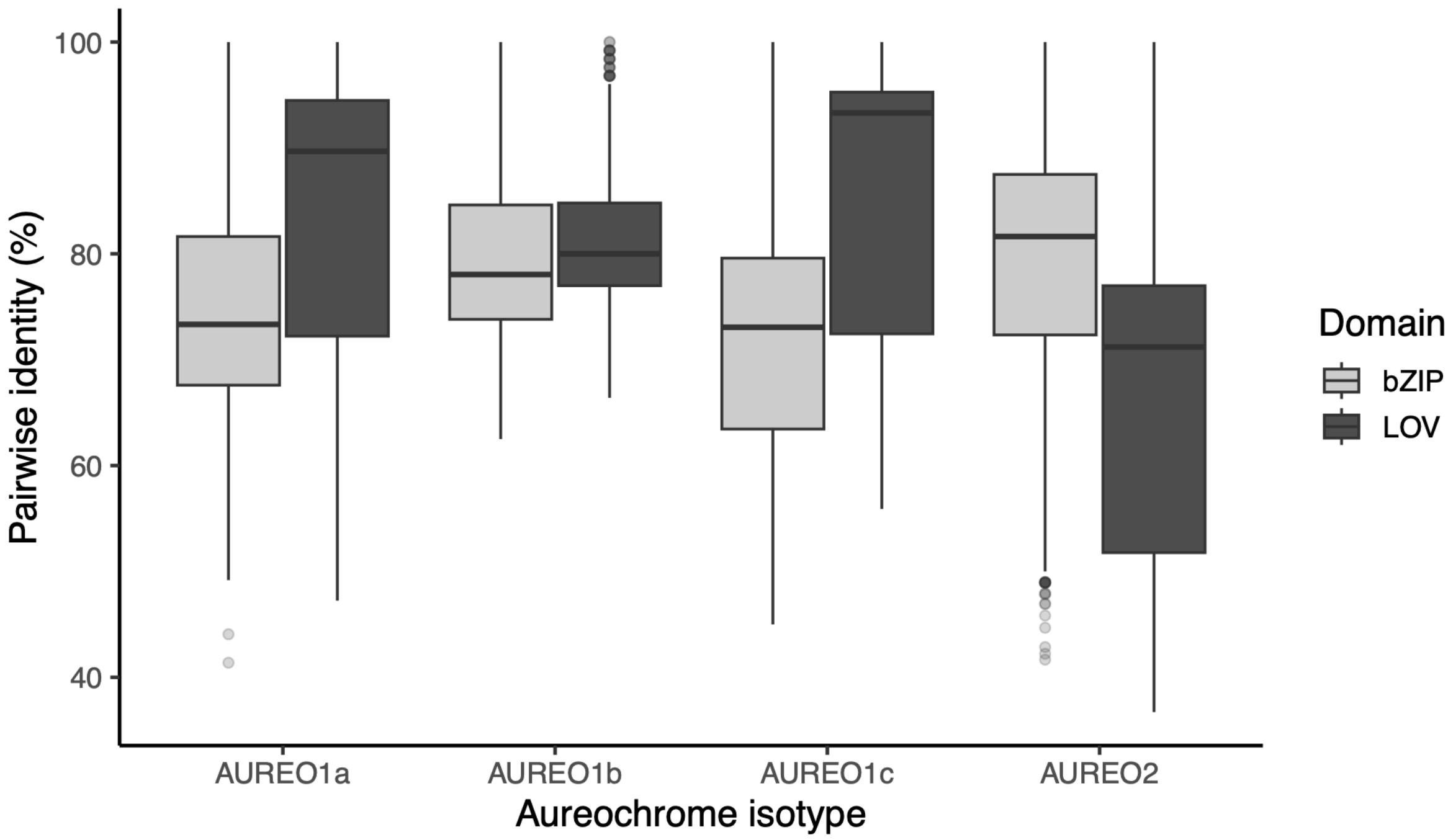

Supplementary Figure 9: Pairwise amino acid identity of AUREO isotypes. Boxplots show the distribution of pairwise amino acid sequence identities (%) for LOV and bZIP domains across AUREO isotypes. Boxes represent the interquartile range (IQR), with the median indicated by the central line. Whiskers extend to 1.5x IQR, and individual pairwise comparisons are shown as jittered points.

Supplementary Method: Co-phylogeny analysis of LOV and bZIP domains

Phylogenetic relationships of the LOV and bZIP domain from AUREO sequences were inferred from precomputed Profileview trees. Tree visualization and concordance analysis were performed in R v4.4.3 using the packages ape and phytools. The two trees were used to construct a co-phylogenetic analysis using the cophylo function, which optimizes tip ordering between trees by allowing rotational optimization of internal nodes (rotate = TRUE, rotate.multi = TRUE) [5]. A table linking corresponding LOV and bZIP sequences was generated based on shared taxon names and used as input for co-phylogenetic mapping.

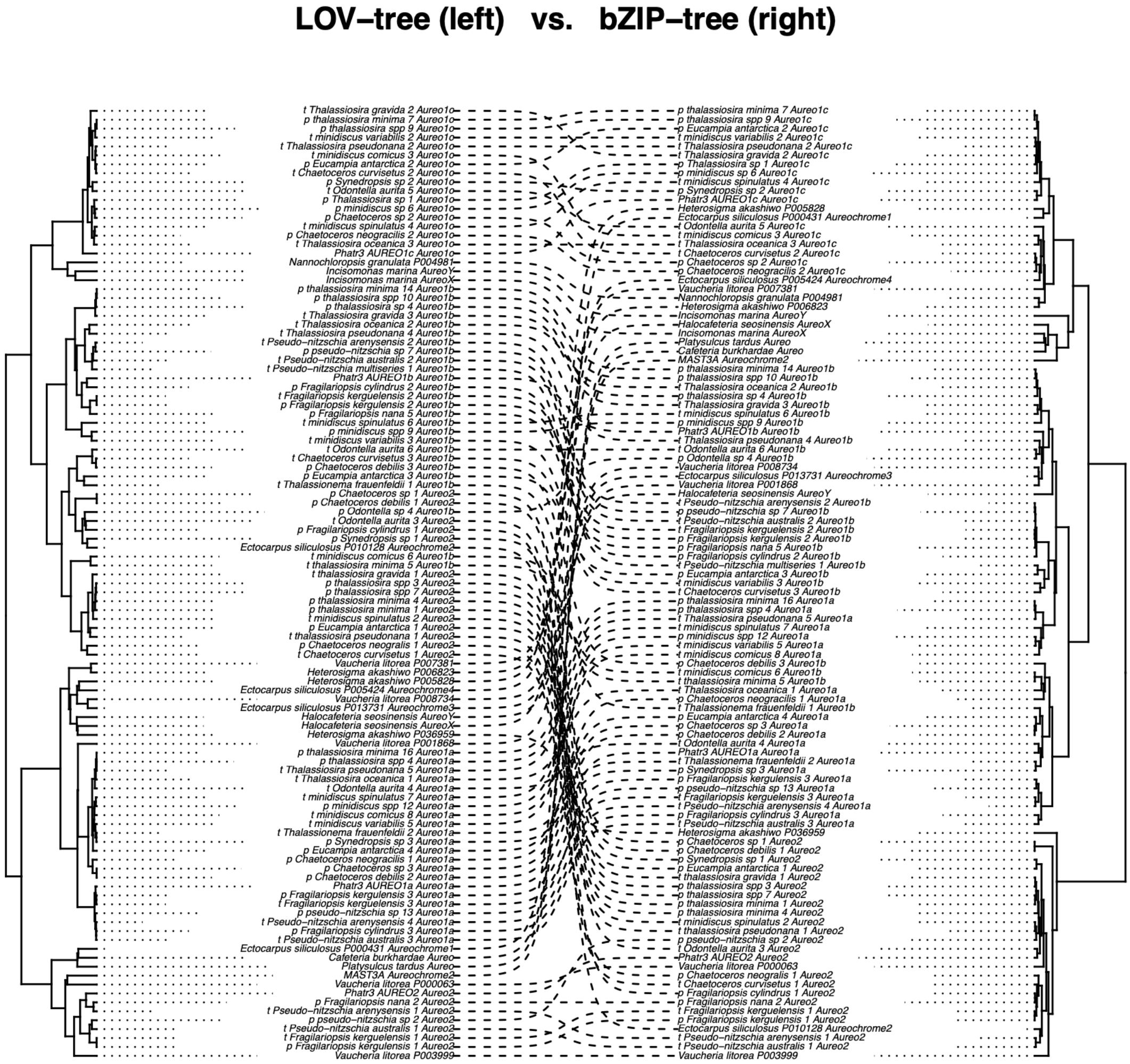

Supplementary Figure 10: Co-phylogenetic comparison of LOV and bZIP domain trees. Co-phylogenetic tanglegram showing the relationship between LOV (left) and bZIP (right) domain phylogenies from AUREO sequences. Tip ordering was optimized using a rotational alignment algorithm to reduce crossing of association links.

Supplementary References

1 Wickham, H. ggplot2: Elegant Graphics for Data Analysis. Springer, Cham, Switzerland (2016). https://ggplot2.tidyverse.org.

2 ggOceanMaps: Plot Data on Oceanographic Maps using 'ggplot2' v. 2.2.0 (2024).

3 Martin, K. *et al*. The biogeographic differentiation of algal microbiomes in the upper ocean from pole to pole. *Nature Communications* **12**, 5483 (2021). https://doi.org/10.1038/s41467-021-25646-9

4 Charif, D. & Lobry, J. R. in *Structural Approaches to Sequence Evolution: Molecules, Networks, Populations* (eds Ugo Bastolla, Markus Porto, H. Eduardo Roman, & Michele Vendruscolo) 207-232 (Springer Berlin Heidelberg, 2007).

5 Revell, L. Phytools: An R package for phylogenetic comparative biology (and other things). *Methods in Ecology and Evolution* **3**, 217-223 (2011). https://doi.org/10.1111/j.2041-210X.2011.00169.x
