# Supplementary material for: Pole-to-pole LOV-domain receptor diversity points to an early stramenopile origin and global dominance of aureochromes
Source: Commun Biol. 2026 Oct 31;9:1464. doi: 10.1038/s42003-026-11067-1 (PMC13737086; doi:10.1038/s42003-026-11067-1)
Supplement: Supplementary file 2 [file 42003_2026_11067_MOESM2_ESM.pdf]

Description of Additional Supplementary Files

Pole-to-pole LOV-domain receptor diversity points to an early stramenopile origin and global dominance of Aureochromes

This file provides descriptions of Supplementary Data 1-8.

File name: Supplementary Data 1
Description: Source Data for Figure 1. Sequence IDs corresponding to the 'Sea of Change' dataset (https://genome.jgi.doe.gov/portal/SeaofArctiOcean/SeaofArctiOcean.info.html) of LOV-domain sequences with their taxonomic origin, station code, and domains included from the 'Sea of Change: Eukaryotic Phytoplankton Communities in the Arctic Ocean' metatranscriptome.

File name: Supplementary Data 2
Description: Source Data for Figure 2. Sequence IDs (EukProt v3 database: https://evocellbio.com/eukprot/) of AUREO sequences used for the phylogenetic and gene duplication analysis in this study, with their assigned taxonomy and the AUREO isoform categorization.

File name: Supplementary Data 3
Description: Source Data for Figure 3 and 4. Sequence IDs of AUREO sequences used for the Profileview analysis with their taxonomic origin, location origin, AUREO isoform, and sequence origin.

File name: Supplementary Data 4
Description: Amino acid sequences of AUREOS used in this study. Publicly available at 10.6084/m9.figshare.33394543.

File name: Supplementary Data 5

Description: Information from the custom reference database collection.

File name: Supplementary Data 6

Description: Unrooted full-length AUREO phylogeny tree file.

File name: Supplementary Data 7 (D1L1)

Description: Root optimization outputs per UFBoot tree for low duplication costs.

File name: Supplementary Data 8 (D1000L1)

Description: Root optimization outputs per UFBoot tree for high duplication costs.
